# Comparison between the crystal structures of racemic and enantiopure aryl benzyl sulfoxides

**DOI:** 10.1039/d5ra06476g

**Published:** 2025-10-09

**Authors:** Maria Annunziata M. Capozzi, Angel Alvarez-Larena, Joan F. Piniella Febrer, Cosimo Cardellicchio

**Affiliations:** a CNR ICCOM, Dipartimento di Chimica, Università di Bari Via Orabona 4 70125 Bari Italy cardellicchio@ba.iccom.cnr.it; b Dipartimento di Chimica, Università di Bari Via Orabona 4 70125 Bari Italy; c Servei de Difracció de Raigs X, Universitat Autònoma de Barcelona 08193 Bellaterra, Cerdanyola del Vallès Barcelona Spain Angel.Alvarez@uab.cat; d Departament de Geologia, Universitat Autònoma de Barcelona 08193 Bellaterra, Cerdanyola del Vallès Barcelona Spain Juan.Piniella@uab.cat

## Abstract

In literature reports about the comparison between the crystal structures of racemic and enantiopure compounds, minor differences have been observed between the conformations of an enantiopure compound and the conformations of the same enantiomer when they are a part of a racemic compound. In the present investigation, the crystal structures of aryl benzyl sulfoxides show peculiar behaviours, in particular when the choice between *anti* or *gauche*-conformers was investigated. In three cases of poly-halogenated molecules, the most frequent *anti*-conformations remain for the crystal structures of racemic compounds, whereas the corresponding enantiopure compounds arrange in *gauche*-conformations. Energy calculations confirm the interactions building up the crystal structures and suggest the reasons for the *gauche*–*anti* choice.

## Abstract 2

Crystal structures of some racemic aryl benzyl sulfoxides arrange in an *anti*-conformation; the corresponding enantiopure sulfoxides arrange in a *gauche*-conformation.

## Graphical abstract

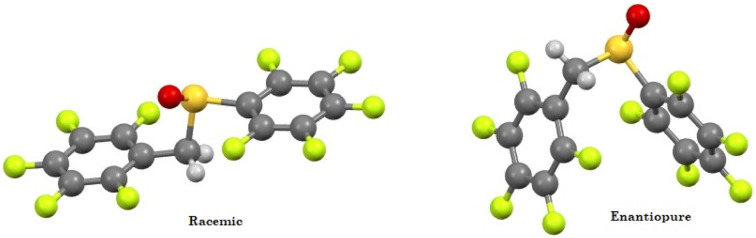

## 1. Introduction

The current definition of crystal engineering embraces both the understanding of intermolecular interactions in the context of crystal packing and the active utilization of this comprehension to design new solids with desired physical and chemical properties.^1^ Many themes merge in this science, one of them being the prediction of crystal structures starting from the molecular formula of a compound.^1^

Alexander I. Kitaigorodski was a pioneer in the comprehension of the interactions between molecules in crystal structures.^2^ His “close packing principle”, in which every molecule is surrounded by the highest number of its peers, is applied especially when van der Waals forces act in the absence of strong electrostatic interactions.^1,2^ For example, aryl hydrocarbons can be satisfactorily described by the close packing principle.^1^ On the other hand, the hydrogen bonding is stronger and directional and allows connections that deviate from close packing. Directional hydrogen bonding and close packing are not excluding because the dispersion phenomena that drive close packing and the hydrogen bonding can cooperate.^3,4^ As an example, the cooperation between hydrogen bonding and aryl stacking interactions was studied and it is representative.^3,4^

The comparison between the crystal structures of the racemic and enantiopure pairs is a research that was scattered in the literature, mainly connected to the investigation of classes of bioactive compounds.^5–9^ For example, a comparison between the crystal structures of racemic and enantiopure compounds was reported for substituted mandelic acids^5^ and for substituted succinic acids.^6^ In some cases, the crystal structures referring to the racemic and enantiopure pair are quite similar.^5–7^ On the other hand, one case was reported in which the arrangements of the molecules in the crystal structures are considered “very different”.^8^

Another interesting topic of investigation in the racemic/enantiopure contrast is connected to the formation of conglomerates.^10–12^ A conglomerate is a “racemic mixture of two enantiomers with each crystal being made up of a single enantiomer”.^10^ These compounds have a relevant interest from an applicative point of view because, at least in principle, a process of separation of enantiomers in a conglomerate can be set up, thus opening the route to the preparation of single enantiomers without resorting to asymmetric synthesis.^10–12^

In the past years, conglomerates appeared to be rare, but recent investigations confirmed that they are not so rare, even if they remain elusively scattered in the crystallographic databases.^11,12^

In our research on the asymmetric oxidation of aryl benzyl sulfides to yield aryl benzyl sulfoxides,^13–19^ we have synthesised a large chemical library constituted by more than 65 enantiopure aryl benzyl sulfoxides, together with their racemic counterparts. Many of the synthesised sulfoxides were suitable for a single crystal X-ray diffraction.^13–19^

In the above chemical library, we first observed that a highly enriched enantiomeric mixture can yield an enantiopure sulfoxide upon crystallisation,^13–19^ and then the intriguing presence of conglomerates.^19^ At this point, we decided to perform a more complete investigation and comparison between the crystal structures of racemic and enantiopure aryl benzyl sulfoxides.

## 2. Results and discussion

We found in the literature the crystal structures of the prototype racemic^20^ and enantiopure^21^ benzyl phenyl sulfoxide 1 (Table 1). We added to this compound the following pairs of crystal structures of racemic and (*R*)-aryl benzyl sulfoxides deriving from our research (Table 1): 4-bromophenyl 2-methoxybenzyl sulfoxide 2;^13,15^ 4-bromophenyl 2-nitrobenzyl sulfoxide 3;^13,15^ 4-bromophenyl 4-nitrobenzyl sulfoxide 4;^13,15^ 4-bromophenyl 3-chlorobenzyl sulfoxide 5;^13,15^ 2,3,4,5,6-pentafluorobenzyl 2,3,4,5,6-pentafluorophenyl sulfoxide 6;^14,16,17^ 2-chloro-5-((2,3,4,5,6-pentafluorophenylsulfinyl)methyl)thiophene 7; 2-(2,3,4,5,6-pentafluorobenzyl)sulfinyl thiophene 8;^19^ 2,4-dichlorophenyl 2,3,4,5,6-pentafluorobenzyl sulfoxide 9.^15^

**Table 1:** Crystal structures of racemic and enantiopure aryl benzyl sulfoxides

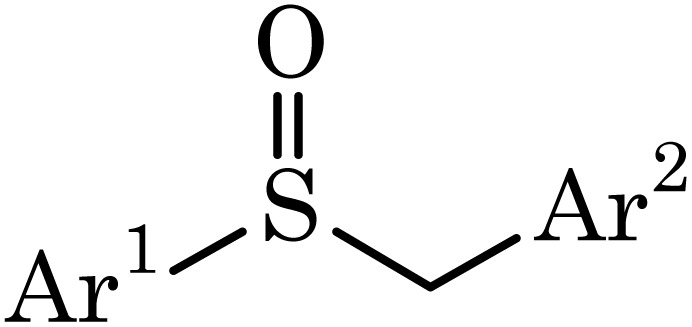								
N	Ar^1^	Ar^2^	*d* a (*rac.*) Mg m^−3^	*d* a (chir.) Mg m^−3^	Torsion^b^ (*rac.*)^c^ (°)	Torsion^b^ (chir.) (°)	Energy^d^ (*rac*) (kJ mol^−1^)	Energy^d^ (chir) (kJ mol^−1^)
1	C_6_H_5_	C_6_H_5_	1.326	1.293	176.3(4)	−174.1(1)	−120.3	−116.9
2	4-Br-C_6_H_4_	2-CH_3_O-C_6_H_4_	1.592	1.586	175.2(2)	179.1(4)	−142.3	−131.0
3	4-Br-C_6_H_4_	2-NO_2_-C_6_H_4_	1.661	1.643	178.9(2)	−166.9(3)	−125.6	−128.5
4	4-Br-C_6_H_4_	4-NO_2_-C_6_H_4_	1.715	1.665	58.6(4)	51.8(5)	−140.0	−124.0
5	4-Br-C_6_H_4_	3-ClC_6_H_4_	1.642	1.643	175.3(2)	52.1(6)	−129.6	−113.9
6	C_6_F_5_	C_6_F_5_	1.970	1.918	173.6(2)	−64.5(4)	−129.2	−124.4
7	C_6_F_5_	2-Cl-(C_4_H_2_S)	1.790	1.808	170.9(3)	67.5(3)	−127.3	−112.9
8	C_4_H_3_S	C_6_F_5_	1.797	1.745	60.9(3)	58.9(3)	−120.4	−111.7
9	2,4-Cl_2_-C_6_H_3_	C_6_F_5_	1.724	1.711	64.1(2)	69.6(6)	−107.4	−105.1
10^e^	2-COOCH_3_-C_6_H_4_	C_6_H_5_	—	1.359	—	62.9(1) 66.0(2)^f^	—	—
11^e^	4-Br-C_6_H_4_	3-CH_3_O-C_6_H_4_	—	1.535	—	55.4(3)	—	—

a *d* = density in Mg m^−3^.

b Torsion angle (in °, see text).

c Only absolute value is reported.

d Lattice energies (Crystal Explorer 21 estimation).

e Conglomerate.

f *Z*′ = 2.

Methyl 2-(benzylsulfinyl)benzoate 10,^13,15,18^ and 4-bromophenyl 3-methoxybenzyl sulfoxide 11,^13^ are peculiar, and will be discussed hereinafter.

The crystal structures of *rac*-1,^20^ (*R*)-1,^21^ (*R*)-2,^13^ (*R*)-3,^13^ (*R*)-4,^13^ (*R*)-5,^13^ (*R*)-8,^19^ and (*R*)-9,^15^ were already reported. The crystal structures of *rac*-2, *rac*-3, *rac*-4, *rac*-5, *rac*-6, (*R*)-6,^17^*rac*-7, (*R*)-7, *rac*-8 and *rac*-9 are reported herein for the first time, determined by single crystal X-ray diffraction experiments. Crystal data and structure refinements of new structures are collected in the SI Section (Tables S1–S10), together with ORTEP plots and packing plots (Fig. S1–S20).

In Table 1, we reported also the calculated densities and the torsion angles for the aryl carbon/sulfur/methylene carbon/aryl carbon sequence.

The lattice energies of each of the 18 crystal structures under investigation were estimated with the Crystal Explorer 21 program (see Experimental section).^24^ The estimation of the lattice energies are reported in Table 1 for each of the 18 structures 1–9. The complete outputs of the calculation for each of the aryl benzyl sulfoxide under investigation are collected in SI (Tables S11–S28). It is evident that the largest contributions to the lattice energy are provided by the molecules that are closest to the central molecule.

The crystal structures of *rac*-10 and *rac*-11 (HPLC checked) were found to be equal to the known structure of (*R*)-10,^13^ and (*R*)-11.^13^ Thus, we had selected a crystal of a single enantiomer from a racemic mixture, an evidence for the formation of a conglomerate. In a previous paper on heterocyclic aryl benzyl sulfoxides,^19^ we reported two conglomerates. Now, we are reporting two further not heterocyclic items to this research, raising the number to 4 conglomerates in the family of the aryl benzyl sulfoxides. This increase has a great relevance, because this family is connected with the blockbuster drug (*S*)-omeprazole.^19,22^ For example, Kellogg *et al.* found a conglomerate in a derivative of omeprazole,^23^ and succeeded in resolving it with the so-called “Viedma ripening”.^10^ To the best of our knowledge, Kellogg's result was not exploited by other groups. We hope that the present report of new conglomerates in this family could stimulate further research.

At this point, we decided to investigate the differences between the crystal structures of enantiopure and racemic sulfoxides 1–9, focusing first on the main interactions observed in the crystal structures and then on the choice between the *gauche*- or *anti*-conformation.

### 2.1. Wallach rule and main interactions

When an equimolar mixture of the (*S*)- and the (*R*)-enantiomers crystallises, there is a strong preference for a pairing between heterochiral molecules, thus yielding racemic crystals, instead of homochiral crystals (in the latter case, a conglomerate is obtained).^25,26^ As a matter of fact, there are by far more racemic crystals than conglomerates in the crystallographic database. Therefore, this preference seems to suggest that racemic crystals pack more efficiently than homochiral crystals,^25^ and consequently that a racemate crystal should be denser than a pure enantiomer crystal (a statement sometimes referred as the “Wallach rule”).^25,27^ This empirical rule is usually fulfilled, even if a careful inspections of the crystallographic database questioned its generality.^25^

In the crystal structures of the investigated aryl benzyl sulfoxides, we found a general confirmation of the Wallach rule, with the exception of sulfoxide 3, in which a calculated slight energy difference favours the enantiopure material (Table 1, entry 3). As far as the density is concerned, the empirical rule was likewise confirmed with the only exception of compounds 5 and 7, but the differences are tiny (Table 1, entries 5 and 7), and it is not safe to draw conclusions based only on these figures.

**Fig. 1:**
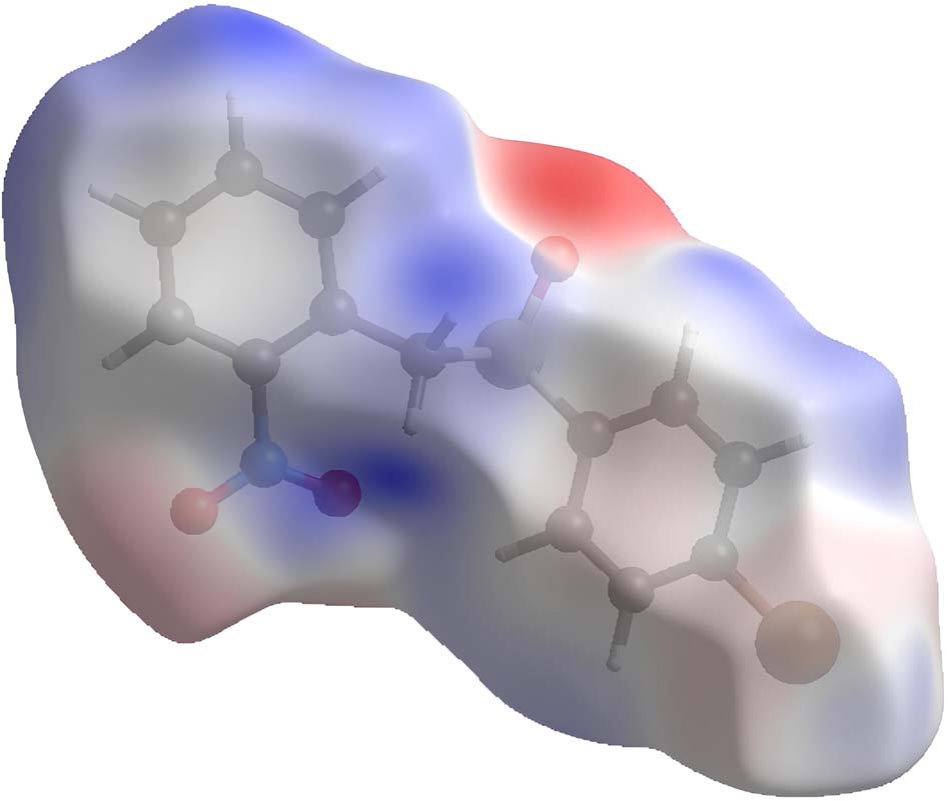
Hirshfeld surface of sulfoxide (*R*)-3 decorated with electrostatic potential (red colour for hydrogen bonding acceptor; blue colour for hydrogen bonding donors. See text).

The main features of the crystal structures of sulfoxides,^20^ and in particular of aryl benzyl sulfoxides,^15^ were already reported. Often, the sulfinyl oxygen atom acts as the hydrogen bonding acceptor. Donors can be one weakly acidic methylene hydrogen atom, or one aryl hydrogen atom. A pictorial representation of this assembly is depicted in Fig. 1, in which the Hirshfeld surface^24^ was decorated with the electrostatic potential,^24^ thus stressing the H-bond donor of the methylene hydrogen atom (in blue colour) and the H-bond acceptor (in red colour) of the sulfinyl oxygen atom in sulfoxide (*R*)-3.

A list of the characteristics of the most relevant hydrogen bondings in sulfoxides reported herein for the first time are collected in Table S29 (SI).

As an exception of this behaviour, the crystal structure of the highly poly-halogenated sulfoxide (*R*)-9 is representative.^15^ No hydrogen bonding was observed, but a network of hydrogen–halogen interactions.^15^

In a previous work of some of us,^28^ weak forms of halogen bonding^29^ were also recognised in two alkyl *p*-bromophenyl sulfoxides. We collected in Table S30 (SI) the characteristics of halogen bonding observed in *rac*-3 and *rac*-4 sulfoxides. In the case of *rac*-3 sulfoxide, the bromine-oxygen intermolecular distance is similar to the values already reported.^28^ On the other hand, *rac*-4 sulfoxide is of particular interest. In fact, the intermolecular contact between the bromine and the oxygen atoms is particularly short (3.04 Å). In a search on the CSD (Cambridge Structural Database. Last release May 2025),^30^ we found that only 4 bromophenyl derivatives have an intermolecular distance of the bromine atom with a sulfinyl oxygen in the 3–3.05 Å range, the most significant of them being the sterically hindered sulfoxide (CSD refcode IFOBIQ) with a 3.00 Å distance.^31^ Moreover, it must be stressed that halogen bonding is absent in the corresponding (*R*)-4 sulfoxide.

**Fig. 2:**
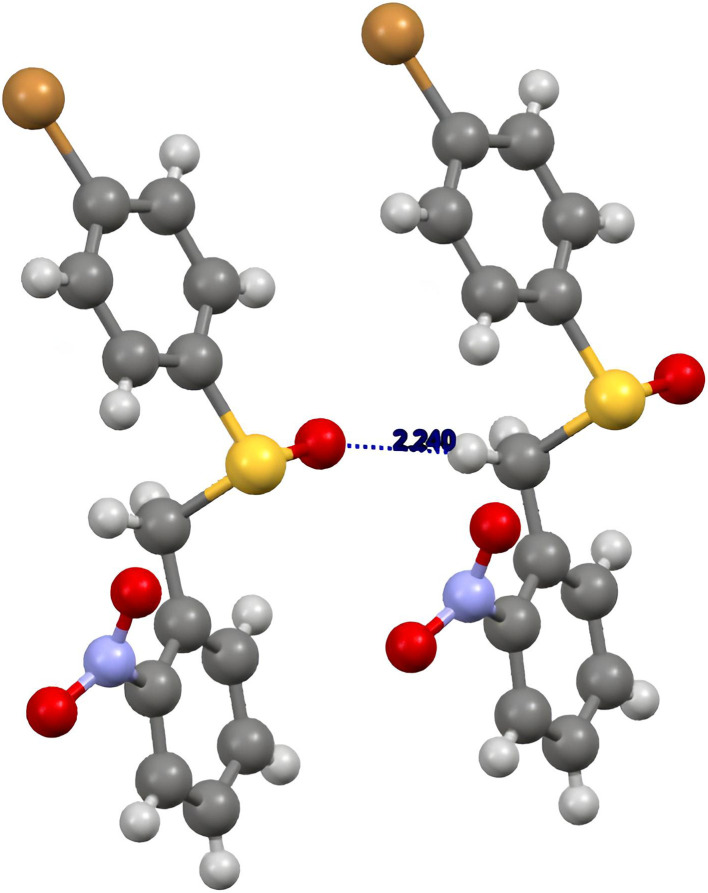
Hydrogen bonding and stacking interactions in sulfoxide (*R*)-3.

In aryl benzyl sulfoxides, weak interactions connected with the presence of aryl groups (*e.g.* aryl stacking) were also recognised.^15^ For example, Fig. 2 describes the interactions building up the crystal structure in sulfoxide (*R*)-3; first, the hydrogen bonding between the sulfinyl oxygen atom and one methylene hydrogen atom, and secondly stacking interactions between the aryl groups.

### 2.2. *Gauche*- or *anti*-conformations

**Fig. 3:**
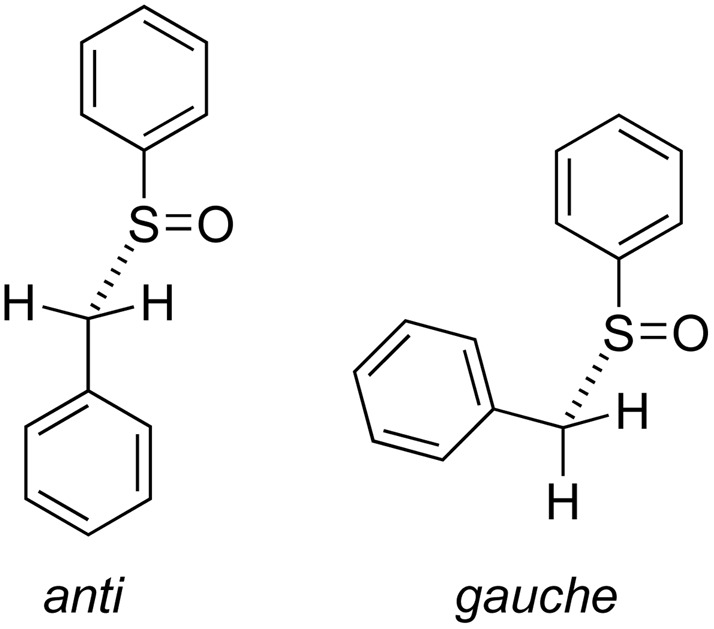
*Anti* or *gauche* conformations for benzyl phenyl sulfoxide 1.

Another relevant topic of investigation is connected to the *anti*- or *gauche*-conformations of the structures of these sulfoxides.^13,15,18^ In Fig. 3, the different conformations for the prototype benzyl phenyl sulfoxide 1 are represented.

In another search on the CSD, if the sulfinyl moiety is not encompassed into a cycle, we found 26 crystal structures of *anti*-conformed aryl benzyl sulfoxides and 13 crystal structures of *gauche*-conformed ones, when duplicates are removed. A prevision of the conformations of crystal structures is not an easy task and it is also one of the factors that renders elusive a general crystal structure prediction.^32,33^ The difficulties arise because the crystallisation process is a delicate balance between many instances.^32,33^

In a previous work of some of us on circular dichroism,^34,35^ the energy differences between the *gauche*- and the *anti*-conformers of some aryl benzyl sulfoxides were calculated. For example, in the case of sulfoxides 3 and 4,^34^ the calculated most stable conformers are the same conformers found in the crystal structures of the present work. However, this fact could be a coincidence, because it is known that flexible molecules can adopt also higher energy conformations in the building up of the crystal structures, when these conformations give rise to more stabilising intermolecular interactions.^32^

In a past paper,^32^ the concepts of “extended” and “compact” conformations were introduced, a description that fits well with the *anti*- and *gauche*-conformations of the present work. In the absence of a strong hydrogen bond, “extended” conformations were considered favourite, due to a larger molecular surface area, that allows more stabilising interactions.

In the investigation on the crystal structures of enantiopure and racemic mandelic acids derivatives,^5^ the need for packing the phenyl groups characterises both the nucleation and the growth of crystals. The presence of different substituents on the aryl groups can alter the overall scaffold of the crystals. In our previous work,^15^ an empirical rule based upon the presence of *ortho*-substituents, that deeply alters the packing of the aryl moieties, can account for the prediction of simple cases. Predictions turn to be difficult when the pentafluorophenyl group is a part of the molecule, and the number of “unexpected” *gauche*-conformations increases among these crystal structures.^15^

In investigations of other research groups,^5–9^ the conformations of the crystal structures of racemic and enantiopure pairs under investigation do not show significant differences. Even when a “large difference” was reported,^8^ the torsion angles found comparing the crystal structure of the enantiopure stereoisomer with the crystal structures of the same enantiomer when it is a part of the racemic crystal are similar. In the present investigation, we observed the same *anti*-conformations among the crystal structures of racemic and enantiopure sulfoxides 1, 2, and 3 (Table 1, entries 1–3). On the other hand, the crystal structures of sulfoxide 4 and the fluorinated sulfoxides 8 and 9 are in a *gauche*-conformation both when they are racemic and when they are enantiopure.

Sulfoxides 5, 6 and 7 are peculiar. The crystal structures of the racemic crystals are in an *anti*-conformation (Table 1, entries 5–7), whereas the crystal structures of the enantiopure crystals are in a *gauche* conformation. The energy calculation for sulfoxide 6,^34^ shows a preference for the *gauche*-conformation, as occurs in the crystal structure of (*R*)-6. At this point, it is not easy to explain the *anti*-conformation found in *rac*-6 crystal structure.

In summary, a simple conformation' prevision based only on conjectures deriving from the aryl groups' substitution, or depending only on thermodynamic factors, is not satisfactory.

Looking for suggestions to uncover the conformations' peculiarity of sulfoxides 5–7, we extracted some pairwise calculations from the data reported in Tables S11–S28 (SI) and we collected them in Table 2. In most cases, we extracted only the pair that gives the largest contribution to the final energy (indicated with I). Sometimes, we added also the second largest contribution (indicated with II, as in entries 2, 7, 14 and 22). In Table 2, we reported only the Crystal Explorer 21 estimated contributions to the total energies provided by the electronic and dispersion energies, the most meaningful interactions.

**Table 2:** Calculated energy lattice data for selected pair of molecules

Entry	N		R^a^ (Å)	*E*_ele_ b (kJ mol^−1^)	*E*_disp_ c (kJ mol^−1^)	*E*_tot_ d (kJ mol^−1^)	SymOp^e^
1	*rac*-1	I^f^	5.47	−18.7	−31.4	−36.1	Displace_b_
2	*rac*-1	II^g^	4.84	−15.3	−36.6	−32.8	Glide
3	(*R*)-1	I^f^	5.67	−18.8	−29.0	−34.2	Displace_a_
4	*rac*-2	I^f^	7.30	−10.2	−27.2	−26.6	Displace_a_
5	(*R*)-2	I^f^	7.78	−5.7	−44.2	−29.3	Displace_c_
6	*rac*-3	I^f^	7.60	−7.5	−36.7	−29.9	Displace_a_
7	*rac*-3	II^g^	7.12	−16.5	−18.0	−24.6	Glide
8	(*R*)-3	I^f^	5.58	−21.2	−35.2	−38.6	Displace_a_
9	*rac*-4	I^f^	5.58	−16.5	−38.4	−36.0	Displace_a_
10	(*R*)-4	I^f^	5.65	−16.2	−37.2	−36.6	Displace_a_
11	*rac*-5	I^f^	5.42	−19.1	−42.8	−40.5	Glide
12	(*R*)-5	I^f^	5.68	−22.9	−37.7	−43.3	Displace_a_
13	*rac*-6	I^f^	5.39	−16.7	−39.1	−40.6	Glide
14	*rac*-6	II^g^	5.38	−7.4	−45.4	−39.5	Displace_b_
15	(*R*)-6	I^f^	5.92	−4.1	−36.6	−29.7	Displace_b_
16	*rac*-7	I^f^	5.14	−15.8	−42.9	−43.0	Displace_b_
17	(*R*)-7	I^f^	5.43	−26.0	−38.6	−45.7	Displace_a_
18	*rac*-8	I^f^	5.35	−20.5	−39.7	−40.2	Displace_b_
19	(*R*)-8	I^f^	5.49	−18.1	−36.4	−38.4	Displace_a_
20	*rac*-9	I^f^	7.89	−15.3	−24.3	−26.1	Displace_a_
21	(*R*)-9	I^f^	6.46	−17.4	−33.5	−38.1	Rot. Axis
22	(*R*)-9	II^g^	7.07	−2.5	−30.5	−21.5	Rot. Axis

a Distance in Å between the centroids of the pairs of molecules under investigation.

b Estimated electronic energy in the pair under investigation.

c Estimated dispersion energy in the pair under investigation.

d Estimated total energy in the pair under investigation as a weighted sum of the various components.

e Type of symmetry operator connecting the pair of the molecules under investigation (see text).

f Highest contributing energy to the lattice energy estimation.

g Second highest contributing energy to the lattice energy estimation.

In the full outputs of the Crystal Explorer 21 calculations (Tables S11–S28), the symmetry operators connecting the molecule in the pair under investigation are reported according to the style of the authors of the program.^24^ In Table 2, we describe only the symmetry operators related to these interactions, adding the terms “Glide” or “Rotation axis” to describe them. The term “Displace” deserves a further explanation. We define as “Displace_a_” the symmetry operator connecting the central molecule with the molecule outside the cell displaced along the [1 0 0] direction. “Displace_b_” and “Displace_c_” are defined likewise along the [0 1 0] and [0 0 1] directions, respectively. It is easy to recognise that the “Displace” operator reported in Table 2 is related to the shortest cell dimensions, as it can be inferred by consulting Table S31 (SI), in which we collected the cell dimensions of the 18 structures of sulfoxides 1–9, taken from the results of this work, or from the literature.^13–16,18–21^

**Fig. 4:**
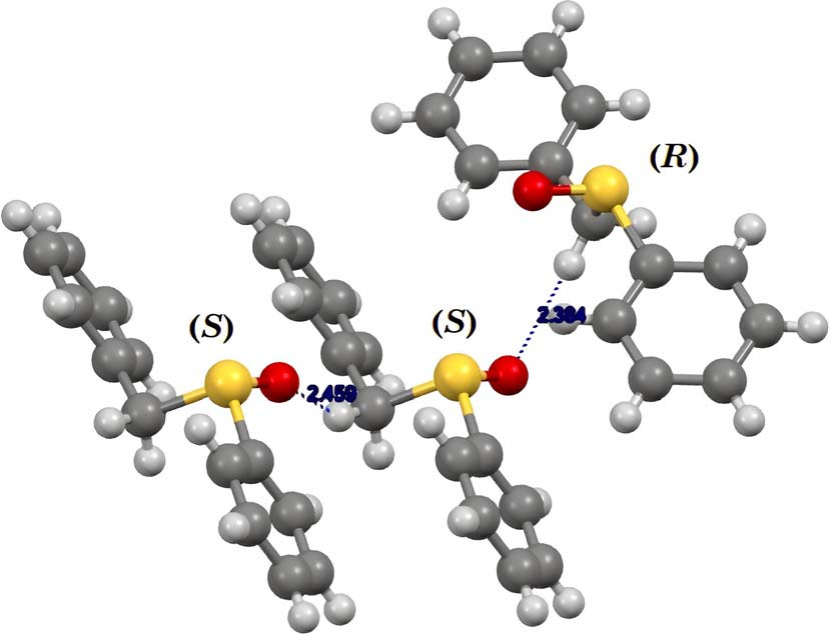
Enantiomers assembly in sulfoxide *rac*-1.

As a representative example of the information that can be recovered from Table 2, we chose the crystal structure of sulfoxide *rac*-1. In Fig. 4, we draw the interactions between the (*R*)- and (*S*)-enantiomers of the racemic crystal. Two (*S*)-enantiomers are connected by the “Displace_b_” operator, due to a weak hydrogen bonding between the oxygen atom and one methylene hydrogen atom. On the other hand, the (*R*)- and the (*S*)-enantiomers are connected by a glide, due to a weak hydrogen bonding between the oxygen atom and the other methylene hydrogen atom (Table S11, SI). The interaction between homochiral enantiomers contributes to the lattice energy for −36.1 kJ mol^−1^ (Table 2, entry 1), whereas the interaction between the two heterochiral enantiomers for −32.8 kJ mol^−1^ (Table 2, entry 2).

It is possible to find other crucial information in Table 2. If a hydrogen bonding is acting, it is easy to recognise it with a larger contribution of the electronic energy to the lattice energy. On the other hand, if the molecules are connected by stacking, or similar interactions connected to the aryl groups or to the fluorine atoms,^15^ a lower contribution to the electronic energy can be observed in Table 2, whereas dispersion energies give a larger contribution (Table 2, entries 5, 6, 14, 15 and 22).

In the large majority of the data of Table 2, the most stabilising interaction (entries 1, 3–6, 8–10, 12, 14–20) connects two homochiral molecules with the “Displace” operator. In entries 13 and 14, related to sulfoxide *rac*-6, the first and the second contribution to the final lattice energy are similar, since they differ only of 1.1 kJ mol^−1^.

These connections between two homochiral molecules with the “Displace” operator are based mainly both on hydrogen bonding and on stacking interactions. In these connections, there is no large difference between the behaviours of *gauche*- and the *anti*-conformations, as it can be inferred by the pictorial representation of the action of the “Displace” operator in the crystal structure of the *anti*-conformed (*R*)-3 (Fig. S21, SI), and on the *gauche*-conformed (*R*)-4 (Fig. S22, SI). This prevalence of the “Displace” operator among the strongest interactions can be explained by the particular assembly of the aryl benzyl sulfoxides, having two aryl groups, the weakly acidic methylene hydrogen and the sulfinyl oxygen atom. This particular assembly can pack efficiently homochiral molecules.

Exceptions in Table 2 are (*R*)-9, whose peculiarity was already discussed^15^ and *rac*-5. In *rac*-5, the shortest cell dimension is greater than 8.7 Å (see SI, Table S31). Within these dimensions, an “inside the cell” interaction is by far more stabilising than an “outside the cell” interaction (SI, Table S19).

Considering the lattice energy calculations reported in Tables S11–S28, we found convenient to define “the inner sphere” and the “outer shell” of molecules around the central one. The “inner sphere” is the sphere that hosts sulfoxides, whose centroids are within 6 Å from the centroids of the central molecule (stressed in Tables S11–S28, SI, with a yellow background). The “outer shell” is the spherical shell that encompasses sulfoxides whose centroids are in the 6–10.1 Å range from the centroids of the central molecule (stressed in Tables S11–S28, SI with a red background). At this stage, we do not consider sulfoxides beyond the 10.1 Å distance away from the centroid of the central molecule.

In Table 3, we define as *N*_1_ the number of molecules (apart from the central one) in the above defined “inner sphere”; *N*_2_ is the number of molecules in the “outer shell”. The total number of molecules in these two shells is comprised between 6 and 9 for the racemic sulfoxides and from 5 to 7 for the enantiopure ones.

**Table 3:** Distribution of molecules in the “inner sphere” and in the “outer” shell and their contributions to the lattice energy

Entry	Compound	*N*_1_ a (<5.9 Å)	Energy_1_^b^ (kJ mol^−1^)	*N*_2_ c (5.9–10.1 Å)	Energy_2_^d^ (kJ mol^−1^)
1	*rac*-1	3	−95.6	3	−2.1
2	(*R*)-1	2	−62.4	4	−43.1
3	*rac*-2	0		8	−102.7
4	(*R*)-2	0		7	−120.2
5	*rac*-3	0		7	−113.7
6	(*R*)-3	1	−38.6	6	−79.7
7	*rac*-4	1	−36	7	−94.6
8	(*R*)-4	1	−36.6	5	−71.2
9	*rac*-5	4	−100.5	4	−6.7
10	(*R*)-5	1	−43.3	5	−55.3
11	*rac*-6	3	−105.5	3	−1.5
12	(*R*)-6	1	−29.7	4	−81.7
13	*rac*-7	3	−103.5	3	−0.6
14	(*R*)-7	1	−45.7	6	−60.1
15	*rac*-8	1	−40.2	8	−75
16	(*R*)-8	1	−38.4	5	−59
17	*rac*-9	0		8	−101.7
18	(*R*)-9	0		6	−97.6

a *N*_1_ is number of molecules apart from the central one in the “inner sphere”(see text).

b Contribution to the lattice energy due to the *N*_1_ molecules in the inner sphere.

c Number of molecules in the “outer shell” (see text).

d Contribution to the lattice energy due to the *N*_2_ molecules in the outer shell.

Sulfoxide *rac*-1 is the prototype (Table 3, entry 1). 3 molecules in the inner sphere contribute for −95.6 kJ mol^−1^ (79% of the total energy of −120.3 kJ mol^−1^, Table 1) for their interaction with the central one. 3 or 4 molecules account for a large contribution to the lattice energies also in *rac*-5 (Table 3, entry 9), *rac*-6 (Table 3, entry 11) and *rac*-7 (Table 3, entry 13).

In the case of the crystal structures of *rac*-2 (Table 3, entry 3), *rac*-3 (Table 3, entry 5), and *rac*-9 (Table 3, entry 17), the shortest cell dimension is larger than the 6 Å, and *N*_1_ is 0.

In the cases of enantiopure sulfoxides, in the prototype molecule (*R*)-1, void of any substituents on the aryl groups, 2 molecules are hosted in the inner sphere apart from the central one (Table 3, entry 2). The interactions with the central molecule are those shown in Fig. 4. It is the only case in which *N*_1_ is 2 in a crystal structure of an enantiopure sulfoxide. In sulfoxides (*R*)-3 (Table 3, entry 6), (*R*)-4 (Table 3, entry 8), (*R*)-5 (Table 3, entry 10), (*R*)-6 (Table 3, entry 12), (*R*)-7 (Table 3, entry 14) and (*R*)-8 (Table 3, entry 16), *N*_1_ is 1, due to the presence of substituents on the aryl groups.^5^ In sulfoxides (*R*)-2 (Table 3, entry 4) and (*R*)-9 (Table 3, entry 18), *N*_1_ is 0.

By a survey of the data of Table 3, and a comparison with the whole data collected in Tables S11–S28, the coupling between two heterochiral molecules provides a larger contribution to the lattice energy, and this fact is a confirmation of the Wallach rule. In this overview, the peculiar halogen bonding of *rac*-4 compound, absent in the corresponding (*R*)-4, confirms the tight packing of a racemic compound in comparison with the enantiopure counterpart.

In the case of the crystal structures of the enantiopure aryl benzyl sulfoxides, there is no heterochiral pairing. Since *N*_1_ is 1 (2 molecules only for (*R*)-1 sulfoxide), the outer shell should be filled by more molecules, in order to provide satisfactory lattice energy. The case of sulfoxide (*R*)-6 is representative. One molecule in the inner sphere contributes for −29.7 kJ mol^−1^ (Table 3, entry 10) due to the interaction with the central molecule; hydrogen bonding provides low contributions to the lattice energy; 4 molecules in the outer shell contribute for −24.6, −22.8, −21.7 and −12.6 kJ mol^−1^ (SI, Table S22), contributions that derive mainly from dispersion energies. At this point, it is likely that a *gauche*-configuration, that is a configuration of a less extended surface area, turns to be useful, because a more compact conformation guarantees that more molecules can be hosted, and can contribute to the final lattice energy, as an application of the Kitaigorodski close packing principle.

## 3. Conclusions

In the present paper, we reported 10 new crystal structures of aryl benzyl sulfoxides that, joined with other 8 similar ones, constitutes a rich chemical library that allowed us a fruitful comparison between the behaviour of crystal structures of enantiopure and racemic compounds. First of all, two further conglomerates, intermediates that could have further useful technological applications, were recognised in this family, that is the family of the blockbuster drug (*S*)-omeprazole.

In a general framework of confirmation of the Wallach rule, the most interesting feature is the conformation of these compounds. In fact, at variance with previous literature reports, we observed three cases in which the crystal structures of the enantiopure compounds are *gauche*, whereas the crystal structures of the racemic mixture have the *anti*-conformation. Moreover, there are also cases of crystal structures of aryl benzyl sulfoxides in which both the racemic and the enantiopure compounds are *gauche*, especially in the case of fluorinated compounds.

Energy calculations uncover possible reasons for this behaviour. According to the Wallach rule, (*R*)- and (*S*) enantiomers pair tightly in the crystal structures of racemic compounds, thus providing a higher contribution to the stabilising energy of the lattice.

In the case of the crystal structures of enantiopure compounds, both the asymmetry caused by the chirality and the presence of substituents on the aryl groups, cause that only one molecule is hosted in the inner sphere apart from the central molecule. In this situation, the outer shell of coordination needs to be filled with more molecules to obtain satisfactory lattice energy. In this regard, more compact molecules, such as the *gauche*-ones, are useful, even if they have less surface area than the *anti*-conformed ones.

## 4. Experimental section

Chemicals were used as received. High resolution mass spectra were determined with a high-performance liquid chromatography ion trap time-of flight (LC-IT-TOF) mass spectrometer by direct infusion of the samples by using methanol as the elution solvent (the samples were previously dissolved in acetonitrile). NMR spectra were recorded on a ^1^H-500 MHz, ^13^C-125 MHz spectrometer. Copies of spectra are collected in SI (Fig. S23–S26).

Racemic and (*R*)-sulfoxides 1, 2, 3, 4, 5, 6, 8, 9 were already reported. Sulfoxide 7 was synthesised in this work. The yield for sulfoxide (*R*)-7 was not optimised. The (*R*)-configuration was attributed to 7, on the basis of the X-ray diffraction experiment that was performed in this work.

### 4.1. Synthesis of not already published compounds

#### 4.1.1. 2-Chloro-5-((2,3,4,5,6-pentafluorophenylthio)methyl)thiophene

2-Chloro-5-((2,3,4,5,6-pentafluorophenylthio)methyl)thiophene was synthesized by adding 0.8 mL of pentafluorothiophenol (6 mmol) to a solution of 0.83 g of potassium carbonate (6 mmol) and 1 g of 2-chloro-5-(chloromethyl)thiophene (6 mmol) in 60 mL of ethanol. The mixture was reacted for 2 hours at room temperature. Usual work up^17–19^ gave a crude mixture that was purified by distillation (Kugelrohr oven temp. 100–105 °C, *p* = 0.1 torr) obtaining 1.4 g of the title product (71% yield). This sulfide solidifies on standing (mp 39–41 °C) and the crystals were found suitable for the X-ray diffraction experiment.

^1^H-NMR (500 MHz, CDCl_3_) 6.66 (d, *J* = 3.8 Hz, 1H), 6.60 (dt, *J* = 3.8, *J* = 0.8 Hz, 1H), 4.18 (s, 2H). ^13^C-NMR (125 MHz, CDCl_3_) 147.6 (dm, *J* = 247 Hz), 141.6 (dm, *J* = 255 Hz), 137.9, 137.7 (dm, *J* = 255 Hz), 130.2, 126.3, 125.8, 107.8 (m), 33.7. HRMS (ESI-TOF), *m*/*z* calcd for C_11_H_3_ClF_5_S_2_ [M–H]^+^ 328.929. Found [M–H]^+^ 328.928.

#### 4.1.2. 2-Chloro-5-((2,3,4,5,6-pentafluorophenylsulfinyl)methyl)thiophene 7

Racemic 7 was obtained by standard MCPBA oxidation of the corresponding sulfide. Mp 128–129 °C (*n*-hexane/ethyl acetate 7 : 3). Sulfoxide (*R*)-7 was obtained according to our standard enantioselective oxidation of the corresponding sulfide with TBHP in *n*-hexane,^13–19^ in the presence of 5 mol% of a complex between titanium *i*-propoxide and (*S*,*S*)-hydrobenzoin. Chromatographic purification yielded 7, having a 76% ee value (ee values were measured with chiral HPLC with Chiralpak IA Column. Eluent: *n*-hexane/*i*-propanol 7 : 3). Yield: 44%. This sample was crystallised from ethanol. We observed that the ee value increased, as often occurred in our work with this type of sulfoxides,^13–19^ and that the crystallised sample was enantiopure (ee >98%). Mp 112–114 °C. The crystals were suitable for the X-ray diffraction experiments.

^1^H-NMR (500 MHz, CDCl_3_) 6.81 (d, *J* = 3.8 Hz, 1H), 6.77 (dt, *J* = 3.8, *J* = 0.8 Hz, 1H), 4.71 (d, *J* = 13.7 Hz, 1H), 4.64 (d, *J* = 13.7 Hz, 1H). ^13^C-NMR (125 MHz, CDCl_3_) 145.5 (dm, *J* = 255 Hz), 143.8 (dm, *J* = 260 Hz), 137.6 (dm, *J* = 260 Hz), 132.0, 129.1, 127.7, 127.0, 116.5 (m), 54.5. HRMS (ESI-TOF), *m*/*z* calcd for C_11_H_3_ClF_5_OS_2_ [M–H]^+^ 344.924. Found [M–H]^+^ 344.926.

The crystal structures of the already reported sulfoxides can be found in the CSD database with the following codes: *rac*-1 (BOYDOJ); (*R*)-1 (SIBXAF); (*R*)-2 (AHECUM); (*R*)-3 (AHECIA); (*R*)-4 (AHECOG); (*R*)-5 (AHEDEX); (*R*)-8 (GUKKUW); (*R*)-9 (DOWQOX).

### 4.2. Molecular pairwise and lattice energy calculations

Starting from the coordinates recorded in the crystallographic files, a central molecule is selected and then a network of adjacent molecules within a 10 Å radius is built. Then, the interaction energy of each molecule of the network with the central species was calculated.^24^ Four different contributions (electronic, polarization, dispersion and repulsion energies) were determined for each interaction. Then, each contribution is suitably weighted and summed up to yield the energy value of each interaction.^24^ Finally, all these energies were summed up to obtain the lattice energy.^24^

### 4.3. X-ray diffraction experiments

Data collection for single crystal X-ray diffraction experiments was performed by using Mo Kα radiation in a Bruker SMART-APEX diffractometer at room temperature, or by using Cu Kα radiation at 219 K in a Bruker D8 Venture diffractometer. An empirical absorption correction was applied (SADABS). Structures were solved by direct methods (SHELXS)^36^ and refined by full-matrix least-squares methods on *F*^2^ for all reflections (SHELXL-2016).^36^ Non-hydrogen atoms were refined anisotropically. Hydrogen atoms bonded to carbon atoms were placed in calculated positions with isotropic displacement parameters fixed at 1.5 (CH_3_) or 1.2 (CH_2_ and CH) times the *U*_eq_ of the corresponding carbon atoms. Crystal data and further refinement details are collected in the SI.

## Conflicts of interest

There are no conflicts to declare.

## Supplementary Material

RA-015-D5RA06476G-s001

RA-015-D5RA06476G-s002

## Data Availability

The data underlying this study are available in the published article and in the supplementary information (SI). Supplementary information: Crystallographic data, CrystalExplorer21 calculations, characteristics of main hydrogen and halogen bondings and spectral data are available. See DOI: https://doi.org/10.1039/d5ra06476g. CCDC 2475115 ((*R*)-6), 2475116 (*rac*-9), 2475117 (*rac*-7), 2475118 (*rac*-4), 2475119 (*rac*-8), 2475120 (*rac*-2), 2475121 ((*R*)-7), 2475122 (*rac*-5), 2475123 (*rac*-6), 2475124 (*rac*-3) contain the supplementary crystallographic data for this paper.^37*a*–*j*^
