# Supplementary material for: Comparison between the crystal structures of racemic and enantiopure aryl benzyl sulfoxides
Source: RSC Adv. 2025 Oct 9;15(45):37824–32. doi: 10.1039/d5ra06476g (PMC12510303; doi:10.1039/d5ra06476g)
Supplement: RA-015-D5RA06476G-s001 [file RA-015-D5RA06476G-s001.pdf]

Supplementary Information (SI) for RSC Advances.
This journal is © The Royal Society of Chemistry 2025

Electronic Supplementary Information for the paper

Comparison between the Crystal Structures of Racemic and Enantiopure Aryl Benzyl Sulfoxides.

Maria Annunziata M. Capozzi,[b]* Angel Alvarez-Larena,[c] Joan F. Piniella Febrer,[d] and Cosimo Cardellicchio.*[a]

(a) CNR ICCOM, Dipartimento di Chimica, Università di Bari, via Orabona 4, 70125 Bari, Italy. E-mail: cardellicchio@ba.iccom.cnr.it

(b) Dipartimento di Chimica, Università di Bari, via Orabona 4, 70125 Bari, Italy.

(c) Servei de Difracció de Raigs X, Universitat Autònoma de Barcelona, 08193 Bellaterra, Cerdanyola del Vallès, Barcelona, Spain.

(d) Departament de Geologia, Universitat Autònoma de Barcelona, 08193 Bellaterra, Cerdanyola del Vallès, Barcelona, Spain.

Index of Electronic Supplementary Information

Tables S1-S10. Crystal data and structure refinements. p. 2-11
Figures S1-S20. ORTEP plots and packing plot p. 12-21
Tables S11-S28. *Crystal Explorer 21* estimates of lattice energies p. 22-39
Table S29. Characteristic of most relevant hydrogen bonding p. 40
Table S30. Characteristic of most relevant halogen bonding p. 40
Table S31. Cell dimensions p. 41
Figure S21-S22: "Displace" operator action in crystal structures p. 42
Figure S23-S26. Graphical report of spectral data p.43-46

Table S1. Crystal data and structure refinement for *rac*-4-bromophenyl 2-methoxybenzyl sulfoxide (2).

Empirical formula	$C_{14}H_{13}BrO_2S$	
Formula weight	325.21	
Temperature	296(2) K	
Wavelength	0.71073 Å	
Crystal system	Triclinic	
Space group	P_{-1}	
Unit cell dimensions	a = 7.3042(5) Å	α = 96.168(1)°.
	b = 7.6512(5) Å	β = 96.341(1)°.
	c = 12.3586(8) Å	γ = 95.461(1)°.
Volume	678.46(8) $Å^3$	
Z	2	
Density (calculated)	1.592 Mg/m^3	
Absorption coefficient	3.174 mm^{-1}	
F(000)	328	
Crystal size	0.55 × 0.32 × 0.20 mm^3	
Theta range for data collection	2.693 to 28.660°.	
Index ranges	-9<=h<=9, -10<=k<=10, -15<=l<=15	
Reflections collected	5399	
Independent reflections	3118 [R(int) = 0.0169]	
Completeness to theta = 25.000°	99.4 %	
Absorption correction	Semi-empirical from equivalents	
Max. and min. transmission	1 and 0.66	
Refinement method	Full-matrix least-squares on F^2	
Data / restraints / parameters	3118 / 0 / 164	
Goodness-of-fit on F^2	1.069	
Final R indices [I>2sigma(I)]	R1 = 0.0332, wR2 = 0.0886	
R indices (all data)	R1 = 0.0401, wR2 = 0.0920	
Largest diff. peak and hole	0.728 and -0.368 e.$Å^{-3}$	

Table S2. Crystal data and structure refinement for *rac*-4-bromophenyl 2-nitrobenzyl sulfoxide (3).

Empirical formula	$C_{13}H_{10}BrNO_3S$	
Formula weight	340.19	
Temperature	296(2) K	
Wavelength	0.71073 Å	
Crystal system	Monoclinic	
Space group	P 2_1/n	
Unit cell dimensions	a = 7.5989(5) Å	α= 90°.
	b = 13.6591(8) Å	β= 100.127(1)°.
	c = 13.3151(8) Å	γ = 90°.
Volume	1360.50(15) $Å^3$	
Z	4	
Density (calculated)	1.661 Mg/m^3	
Absorption coefficient	3.177 mm^{-1}	
F(000)	680	
Crystal size	0.52 × 0.34 × 0.19 mm^3	
Theta range for data collection	2.153 to 28.761°.	
Index ranges	-9<=h<=9, -18<=k<=18, -17<=l<=17	
Reflections collected	10688	
Independent reflections	3281 [R(int) = 0.0199]	
Completeness to theta = 25.000°	99.9 %	
Absorption correction	Semi-empirical from equivalents	
Max. and min. transmission	1 and 0.70	
Refinement method	Full-matrix least-squares on F^2	
Data / restraints / parameters	3281 / 0 / 172	
Goodness-of-fit on F^2	1.016	
Final R indices [I>2sigma(I)]	R1 = 0.0435, wR2 = 0.1114	
R indices (all data)	R1 = 0.0619, wR2 = 0.1222	
Largest diff. peak and hole	0.731 and -0.328 e.$Å^{-3}$	

Table S3. Crystal data and structure refinement for *rac*-4-bromophenyl 4-nitrobenzyl sulfoxide (4).

Empirical formula	$C_{13}H_{10}BrNO_3S$	
Formula weight	340.19	
Temperature	219(2) K	
Wavelength	1.54178 Å	
Crystal system	Monoclinic	
Space group	P 2_1/c	
Unit cell dimensions	a = 5.5795(3) Å	α= 90°.
	b = 15.8279(7) Å	β= 97.030(3)°.
	c = 15.0311(6) Å	γ = 90°.
Volume	1317.44(11) $Å^3$	
Z	4	
Density (calculated)	1.715 Mg/m^3	
Absorption coefficient	5.778 mm^{-1}	
F(000)	680	
Crystal size	0.080 × 0.060 × 0.060 mm^3	
Theta range for data collection	4.072 to 68.405°.	
Index ranges	-6<=h<=6, -19<=k<=18, -18<=l<=17	
Reflections collected	22469	
Independent reflections	2419 [R(int) = 0.0964]	
Completeness to theta = 25.000°	99.1 %	
Absorption correction	Semi-empirical from equivalents	
Max. and min. transmission	0.72 and 0.55	
Refinement method	Full-matrix least-squares on F^2	
Data / restraints / parameters	2419 / 0 / 172	
Goodness-of-fit on F^2	1.070	
Final R indices [I>2sigma(I)]	R1 = 0.0485, wR2 = 0.1202	
R indices (all data)	R1 = 0.0634, wR2 = 0.1299	
Extinction coefficient	n/a	
Largest diff. peak and hole	0.625 and -1.010 e.$Å^{-3}$	

Table S4. Crystal data and structure refinement for *rac*-4-bromophenyl 3-chlorobenzyl sulfoxide (5).

Empirical formula	$C_{13}H_{10}BrClOS$	
Formula weight	329.63	
Temperature	296(2) K	
Wavelength	0.71073 Å	
Crystal system	Monoclinic	
Space group	P 2_1/c	
Unit cell dimensions	a = 14.7677(9) Å	α= 90°.
	b = 10.6376(7) Å	β= 104.795(1)°.
	c = 8.7815(5) Å	γ = 90°.
Volume	1333.77(14) $Å^3$	
Z	4	
Density (calculated)	1.642 Mg/m^3	
Absorption coefficient	3.418 mm^{-1}	
F(000)	656	
Crystal size	0.41 × 0.35 × 0.11 mm^3	
Theta range for data collection	1.426 to 28.743°.	
Index ranges	-19<=h<=19, -14<=k<=14, -11<=l<=11	
Reflections collected	10355	
Independent reflections	3231 [R(int) = 0.0316]	
Completeness to theta = 25.000°	99.9 %	
Absorption correction	Semi-empirical from equivalents	
Max. and min. transmission	1 and 0.67	
Refinement method	Full-matrix least-squares on F^2	
Data / restraints / parameters	3231 / 0 / 154	
Goodness-of-fit on F^2	1.020	
Final R indices [I>2sigma(I)]	R1 = 0.0412, wR2 = 0.1076	
R indices (all data)	R1 = 0.0609, wR2 = 0.1179	
Largest diff. peak and hole	0.652 and -0.408 e.$Å^{-3}$	

Table S5. Crystal data and structure refinement for *rac*-2, 3, 4, 5, 6-pentafluorobenzyl 2, 3, 4, 5, 6-pentafluorophenyl sulfoxide (6).

Empirical formula	$C_{13}H_2F_{10}OS$	
Formula weight	396.21	
Temperature	296(2) K	
Wavelength	0.71073 Å	
Crystal system	Monoclinic	
Space group	P c	
Unit cell dimensions	a = 12.966(3) Å	α= 90°.
	b = 5.3774(11) Å	β= 93.82(3)°.
	c = 9.6002(19) Å	γ = 90°.
Volume	667.9(2) $Å^3$	
Z	2	
Density (calculated)	1.970 Mg/m^3	
Absorption coefficient	0.368 mm^{-1}	
F(000)	388	
Crystal size	0.670 × 0.420 × 0.400 mm^3	
Theta range for data collection	1.574 to 28.513°.	
Index ranges	-17<=h<=17, -7<=k<=7, -12<=l<=12	
Reflections collected	4991	
Independent reflections	2965 [R(int) = 0.0140]	
Completeness to theta = 25.000°	99.9 %	
Absorption correction	Semi-empirical from equivalents	
Max. and min. transmission	1 and 0.7854	
Refinement method	Full-matrix least-squares on F^2	
Data / restraints / parameters	2965 / 2 / 227	
Goodness-of-fit on F^2	1.078	
Final R indices [I>2sigma(I)]	R1 = 0.0376, wR2 = 0.1048	
R indices (all data)	R1 = 0.0399, wR2 = 0.1069	
Absolute structure parameter	0.22(11)	
Extinction coefficient	n/a	
Largest diff. peak and hole	0.454 and -0.193 e.$Å^{-3}$	

Table S6. Crystal data and structure refinement for (*R*)-2, 3, 4, 5, 6-pentafluorobenzyl 2, 3, 4, 5, 6-pentafluorophenyl sulfoxide (6).

Empirical formula	$C_{13}H_2F_{10}OS$	
Formula weight	396.21	
Temperature	296(2) K	
Wavelength	0.71073 Å	
Crystal system	Monoclinic	
Space group	P 2_1	
Unit cell dimensions	a = 9.0321(9) Å	α= 90°.
	b = 5.9155(6) Å	β= 108.758(3)°.
	c = 13.5634(14) Å	γ = 90°.
Volume	686.19(12) $Å^3$	
Z	2	
Density (calculated)	1.918 Mg/m^3	
Absorption coefficient	0.359 mm^{-1}	
F(000)	388	
Crystal size	0.400 × 0.290 × 0.070 mm^3	
Theta range for data collection	1.586 to 28.541°.	
Index ranges	-11<=h<=11, -7<=k<=7, -18<=l<=18	
Reflections collected	5431	
Independent reflections	3119 [R(int) = 0.0206]	
Completeness to theta = 25.000°	99.7 %	
Absorption correction	Semi-empirical from equivalents	
Max. and min. transmission	1 and 0.7636	
Refinement method	Full-matrix least-squares on F^2	
Data / restraints / parameters	3119 / 1 / 226	
Goodness-of-fit on F^2	1.012	
Final R indices [I>2sigma(I)]	R1 = 0.0441, wR2 = 0.1047	
R indices (all data)	R1 = 0.0608, wR2 = 0.1141	
Absolute structure parameter	0.11(6)	
Extinction coefficient	n/a	
Largest diff. peak and hole	0.248 and -0.173 e.$Å^{-3}$	

Table S7. Crystal data and structure refinement for *rac*-2-chloro-5-((2, 3, 4, 5, 6-pentafluorophenylsulfinyl)methyl)thiophene (7).

Empirical formula	$C_{11}H_4ClF_5OS_2$	
Formula weight	346.71	
Temperature	296(2) K	
Wavelength	0.71073 Å	
Crystal system	Monoclinic	
Space group	P 2_1/c	
Unit cell dimensions	a = 26.479(3) Å	α= 90°.
	b = 5.1357(4) Å	β= 92.4010(10)°.
	c = 9.4706(8) Å	γ = 90°.
Volume	1286.8(2) $Å^3$	
Z	4	
Density (calculated)	1.790 Mg/m^3	
Absorption coefficient	0.672 mm^{-1}	
F(000)	688	
Crystal size	0.480 × 0.460 × 0.080 mm^3	
Theta range for data collection	1.539 to 28.544°.	
Index ranges	-33<=h<=34, -6<=k<=6, -12<=l<=12	
Reflections collected	9487	
Independent reflections	3047 [R(int) = 0.0365]	
Completeness to theta = 25.000°	99.7 %	
Refinement method	Full-matrix least-squares on F^2	
Data / restraints / parameters	3047 / 0 / 181	
Goodness-of-fit on F^2	1.055	
Final R indices [I>2sigma(I)]	R1 = 0.0661, wR2 = 0.1601	
R indices (all data)	R1 = 0.0888, wR2 = 0.1729	
Extinction coefficient	n/a	
Largest diff. peak and hole	0.560 and -0.386 e.$Å^{-3}$	

Table S8. Crystal data and structure refinement for (*R*)-2-chloro-5-((2, 3, 4, 5, 6-pentafluorophenylsulfinyl)methyl)thiophene (7).

Empirical formula	$C_{11}H_4ClF_5OS_2$	
Formula weight	346.71	
Temperature	296(2) K	
Wavelength	0.71073 Å	
Crystal system	Monoclinic	
Space group	$P\ 2_1$	
Unit cell dimensions	a = 5.4298(5) Å	α= 90°.
	b = 9.9501(8) Å	β= 97.2490(10)°.
	c = 11.8841(10) Å	γ = 90°.
Volume	636.93(9) $Å^3$	
Z	2	
Density (calculated)	1.808 Mg/m^3	
Absorption coefficient	0.679 mm^{-1}	
F(000)	344	
Crystal size	0.930 × 0.080 × 0.050 mm^3	
Theta range for data collection	1.727 to 28.732°.	
Index ranges	-7<=h<=7, -12<=k<=12, -15<=l<=16	
Reflections collected	5088	
Independent reflections	2791 [R(int) = 0.0219]	
Completeness to theta = 25.000°	99.6 %	
Absorption correction	Semi-empirical from equivalents	
Max. and min. transmission	1 and 0.870	
Refinement method	Full-matrix least-squares on F^2	
Data / restraints / parameters	2791 / 7 / 181	
Goodness-of-fit on F^2	1.035	
Final R indices [I>2sigma(I)]	R1 = 0.0367, wR2 = 0.0826	
R indices (all data)	R1 = 0.0456, wR2 = 0.0870	
Absolute structure parameter	0.04(4)	
Extinction coefficient	n/a	
Largest diff. peak and hole	0.243 and -0.168 $e.Å^{-3}$	

Table S9. Crystal data and structure refinement for *rac*-2-(2, 3, 4, 5, 6-pentafluorobenzyl)sulfinyl thiophene (8).

Empirical formula	$C_{11}H_5F_5OS_2$	
Formula weight	312.27	
Temperature	219(2) K	
Wavelength	1.54178 Å	
Crystal system	Monoclinic	
Space group	P 2_1/c	
Unit cell dimensions	a = 8.5319(3) Å	α= 90°.
	b = 5.3467(2) Å	β= 97.512(2)°.
	c = 25.5242(8) Å	γ = 90°.
Volume	1154.36(7) $Å^3$	
Z	4	
Density (calculated)	1.797 Mg/m^3	
Absorption coefficient	4.756 mm^{-1}	
F(000)	624	
Crystal size	0.020 × 0.020 × 0.010 mm^3	
Theta range for data collection	3.493 to 65.179°.	
Index ranges	-10<=h<=10, -5<=k<=6, -30<=l<=30	
Reflections collected	32745	
Independent reflections	1971 [R(int) = 0.1274]	
Completeness to theta = 25.000°	100.0 %	
Absorption correction	Semi-empirical from equivalents	
Refinement method	Full-matrix least-squares on F^2	
Data / restraints / parameters	1971 / 0 / 172	
Goodness-of-fit on F^2	1.042	
Final R indices [I>2sigma(I)]	R1 = 0.0434, wR2 = 0.1081	
R indices (all data)	R1 = 0.0593, wR2 = 0.1186	
Extinction coefficient	n/a	
Largest diff. peak and hole	0.392 and -0.513 e.$Å^{-3}$	

Table S10. Crystal data and structure refinement for *rac*- 2,4-dichlorophenyl 2, 3, 4, 5, 6-pentafluorobenzyl sulfoxide (9).

Empirical formula	$C_{13}H_5Cl_2F_5OS$	
Formula weight	375.13	
Temperature	296(2) K	
Wavelength	0.71073 Å	
Crystal system	Monoclinic	
Space group	$P\ 2_1/n$	
Unit cell dimensions	a = 7.8924(7) Å	α= 90°.
	b = 12.8239(11) Å	β= 96.73(3)°.
	c = 14.3783(12) Å	γ = 90°.
Volume	1445.2 (2) $Å^3$	
Z	4	
Density (calculated)	1.724 Mg/m^3	
Absorption coefficient	0.645 mm^{-1}	
F(000)	744	
Crystal size	0.270 × 0.140 × 0.040 mm^3	
Theta range for data collection	2.135 to 28.762°.	
Index ranges	-10<=h<=10, -17<=k<=16, -18<=l<=18	
Reflections collected	11368	
Independent	3507 [R(int) = 0.0471]	
Completeness to theta = 25.000°	100 %	
Absorption correction	Semi-empirical from equivalents	
Max. and min. transmission	1 and 0.788	
Refinement method	Full-matrix least-squares on F^2	
Data / restraints / parameters	3507 / 0 / 199	
Goodness-of-fit on F^2	1.017	
Final R indices [I>2sigma(I)]	R1 = 0.0519, wR2 = 0.1124	
R indices (all data)	R1 = 0.1099, wR2 = 0.1339	
Absolute structure parameter	0.04(4)	
Extinction coefficient	n/a	
Largest diff. peak and hole	0.243 and -0.268 e.$Å^{-3}$	

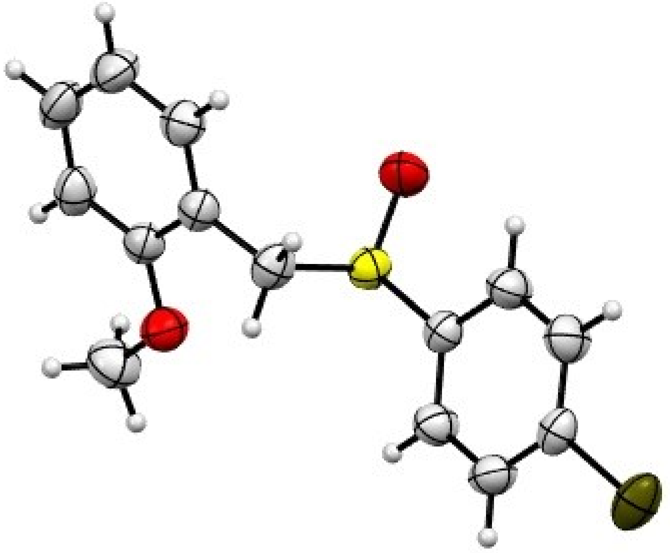

Figure S1. ORTEP plot (50% probability level) of *rac*-4-bromophenyl 2-methoxybenzyl sulfoxide **2**

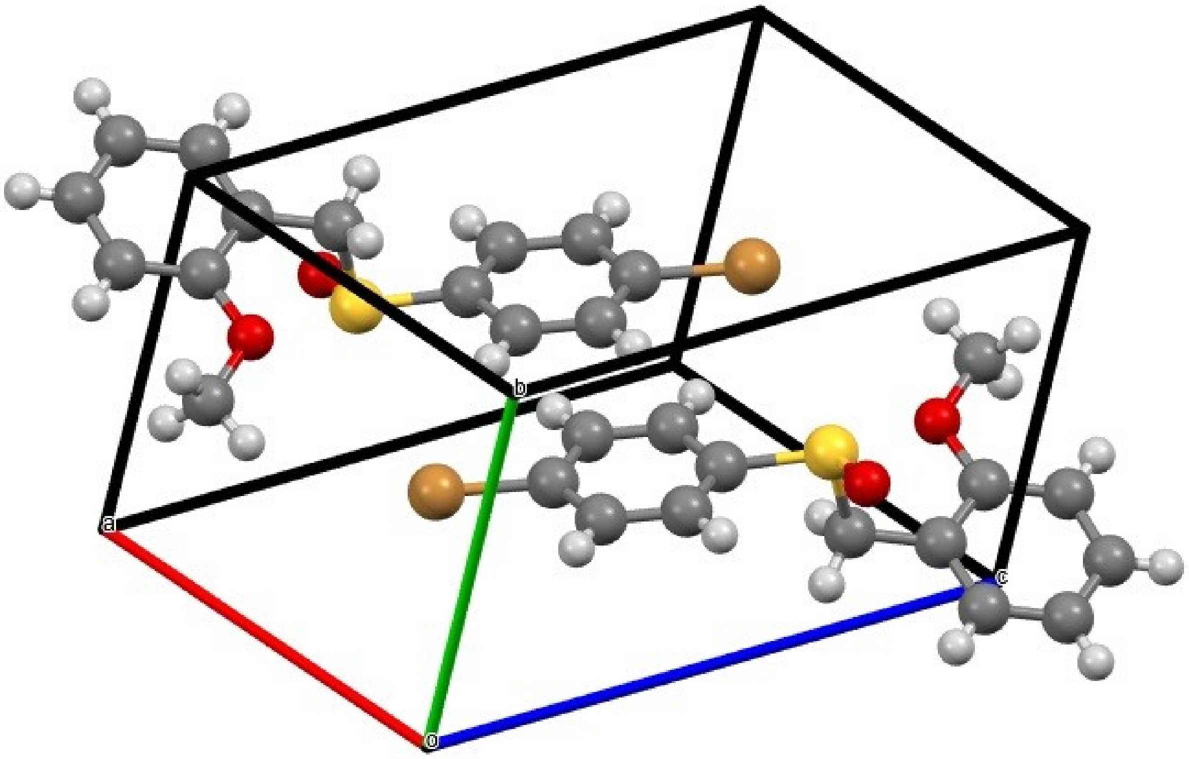

Figure S2. Packing plot of *rac*-4-bromophenyl 2-methoxybenzyl sulfoxide **2**

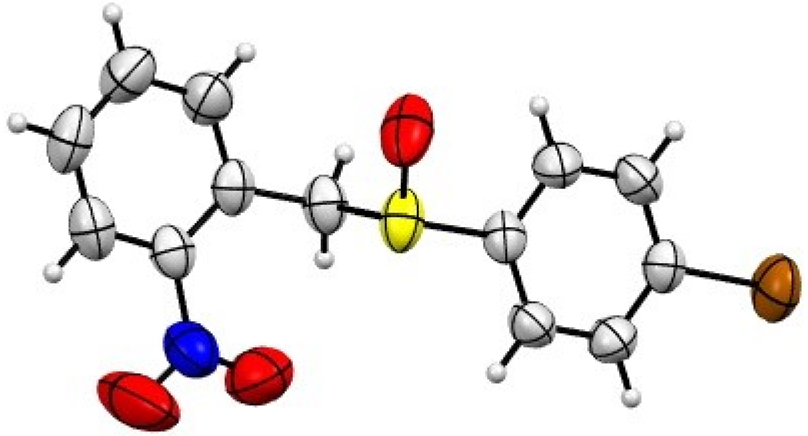

Figure S3. ORTEP plot (50% probability level) of *rac*-4-bromophenyl 2-nitrobenzyl sulfoxide **3**

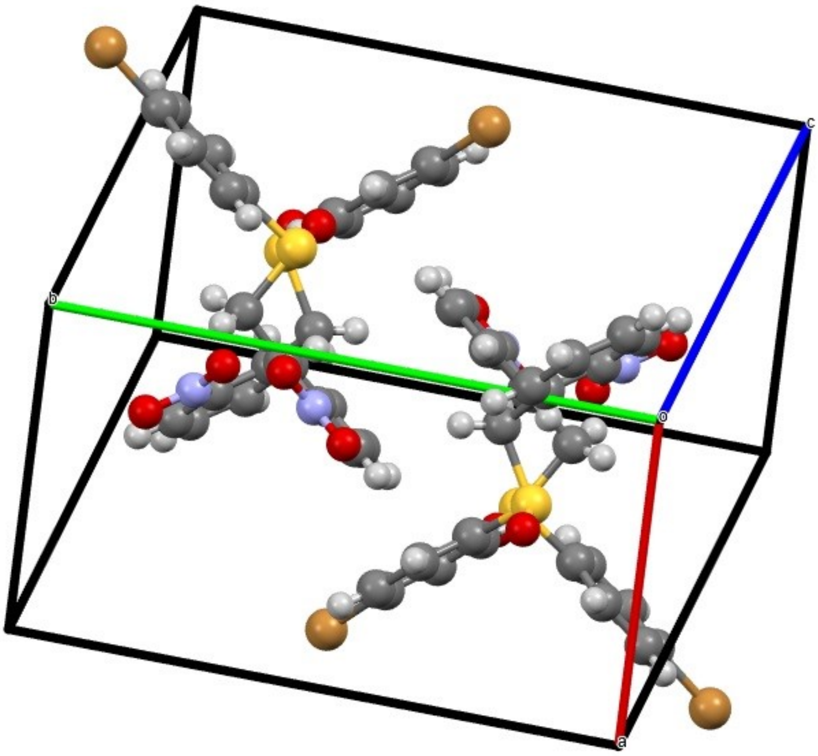

Figure S4. Packing plot of *rac*-4-bromophenyl 2-nitrobenzyl sulfoxide **3**

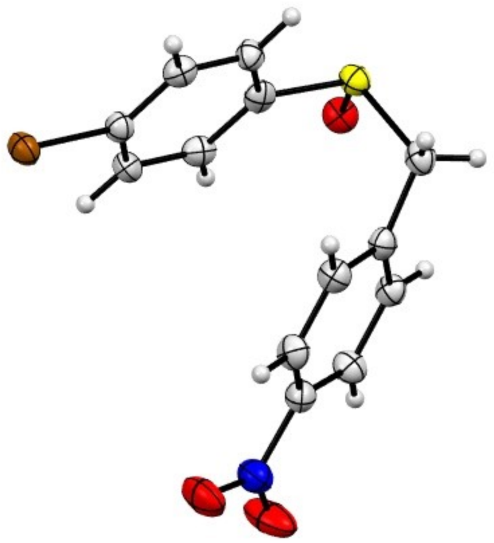

Figure S5. ORTEP plot (50% probability level) of *rac*-4-bromophenyl 4-nitrobenzyl sulfoxide **4**

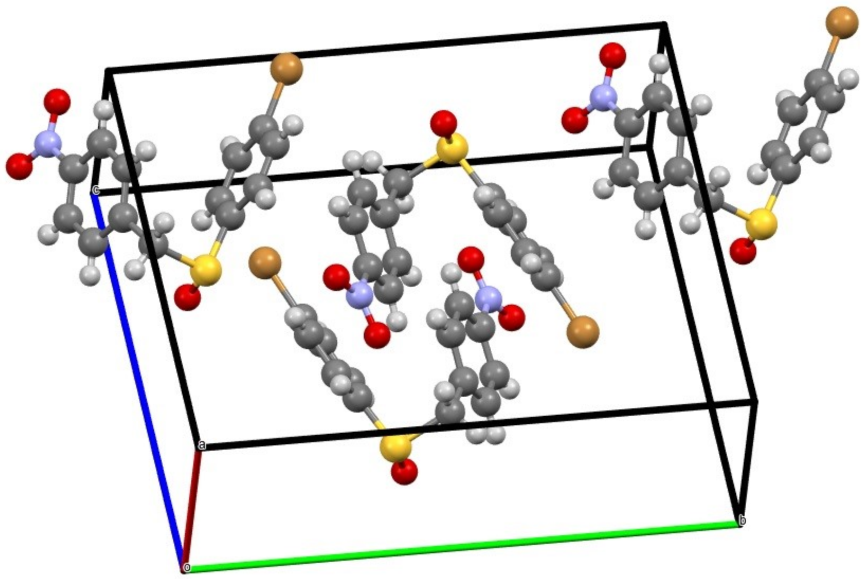

Figure S6. Packing plot of *rac*-4-bromophenyl 4-nitrobenzyl sulfoxide **4**

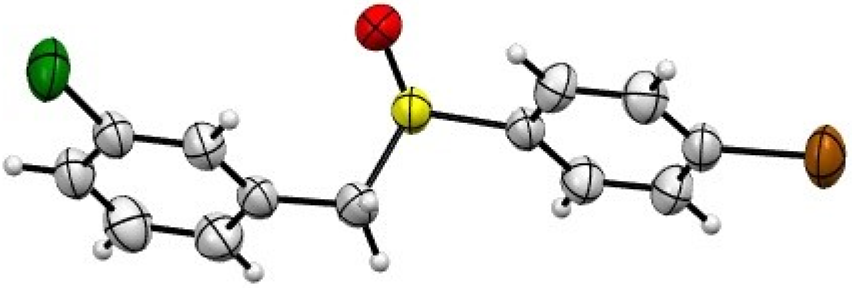

Figure S7. ORTEP plot (50% probability level) of *rac*-4-bromophenyl 3-chlorobenzyl sulfoxide **5**.

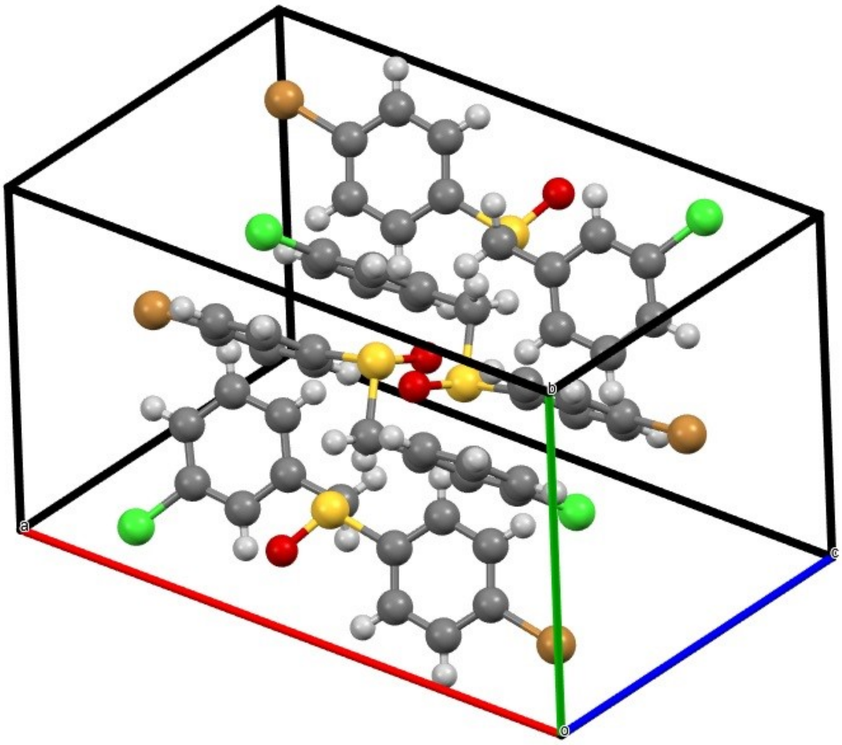

Figure S8. Packing plot of *rac*-4-bromophenyl 3-chlorobenzyl sulfoxide **5**.

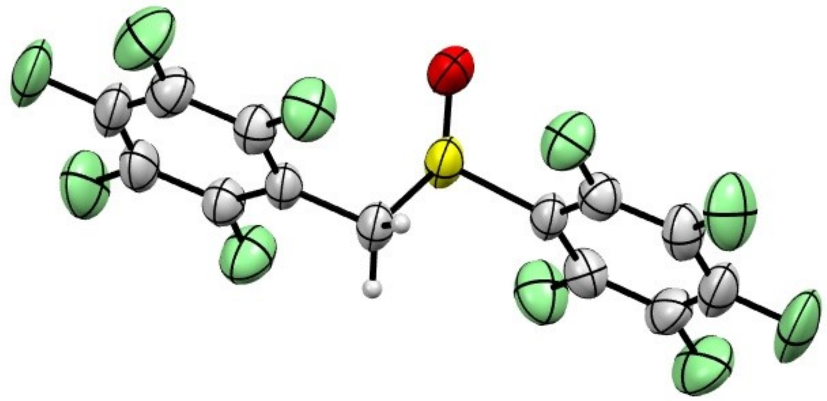

Figure S9. ORTEP plot (50% probability level) of *rac*-2, 3, 4, 5, 6-pentafluorobenzyl 2, 3, 4, 5, 6-pentafluorophenyl sulfoxide **6**

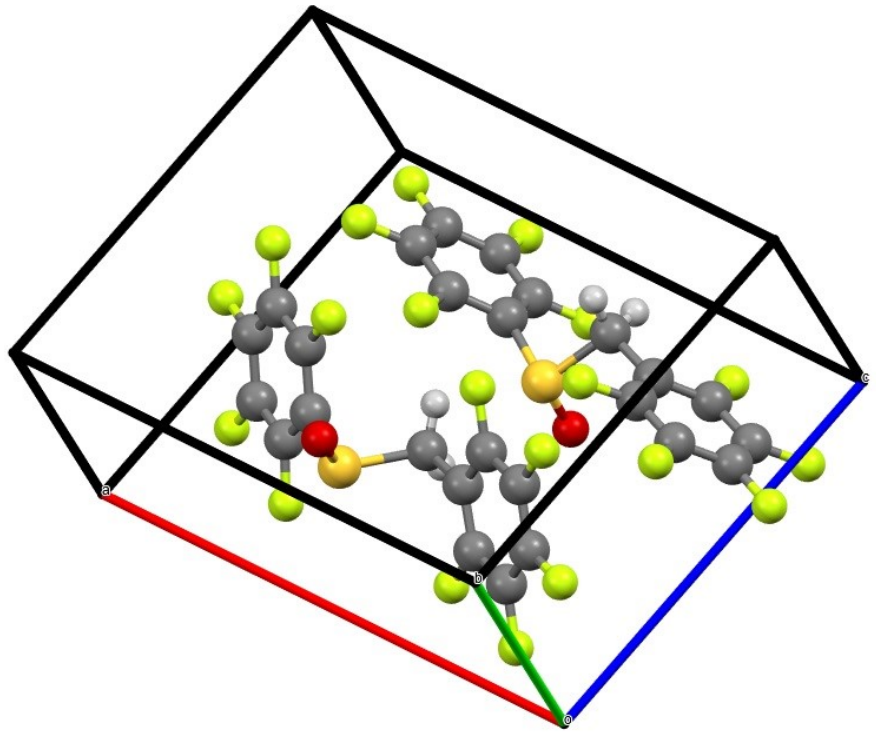

Figure S10. Packing plot of *rac*-2, 3, 4, 5, 6-pentafluorobenzyl 2, 3, 4, 5, 6-pentafluorophenyl sulfoxide **6**.

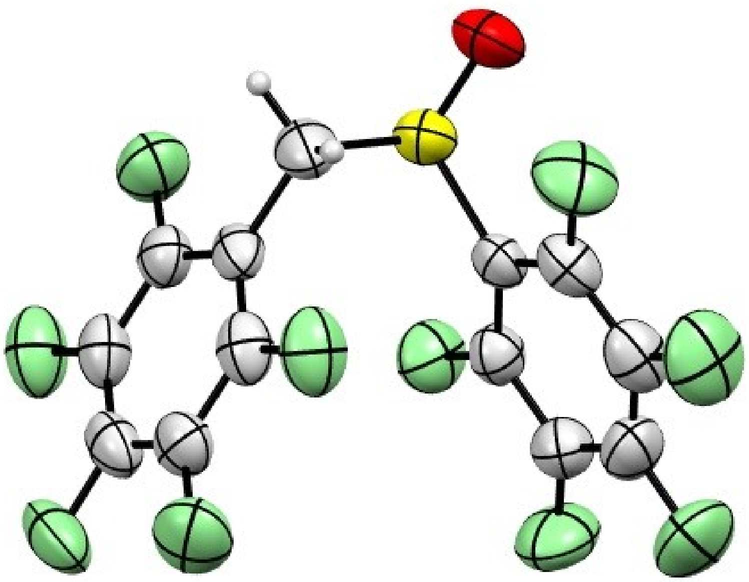

Figure S11. ORTEP plot (50% probability level) of (*R*)-2, 3, 4, 5, 6-pentafluorobenzyl 2, 3, 4, 5, 6-pentafluorophenyl sulfoxide **6**

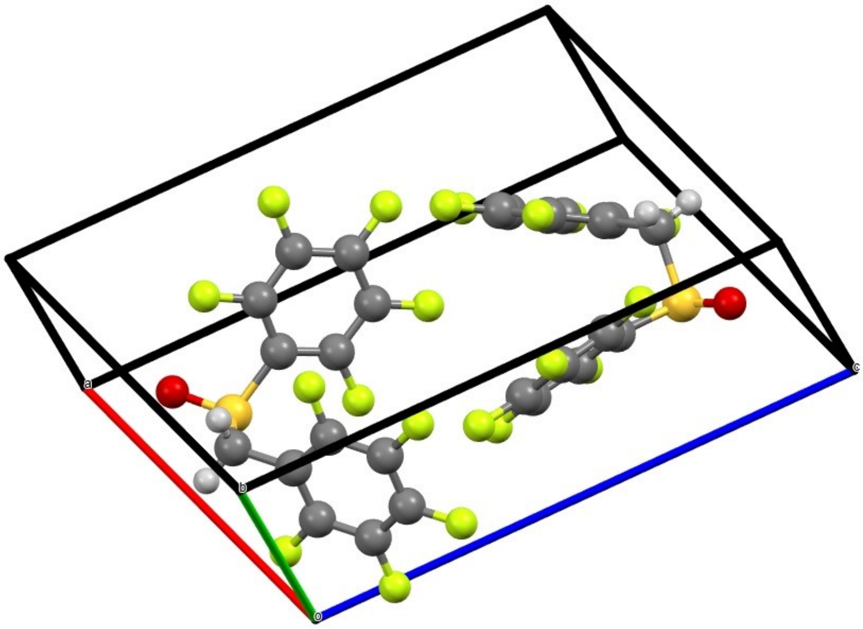

Figure S12. Packing plot of (*R*)-2, 3, 4, 5, 6-pentafluorobenzyl 2, 3, 4, 5, 6-pentafluorophenyl sulfoxide **6**.

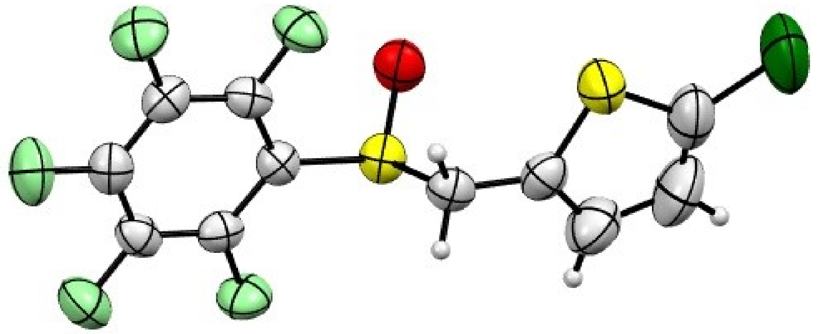

Figure S13. ORTEP plot (50% probability level) of *rac*-2-chloro-5-((2, 3, 4, 5, 6-pentafluorophenylsulfinyl)methyl)thiophene **7**

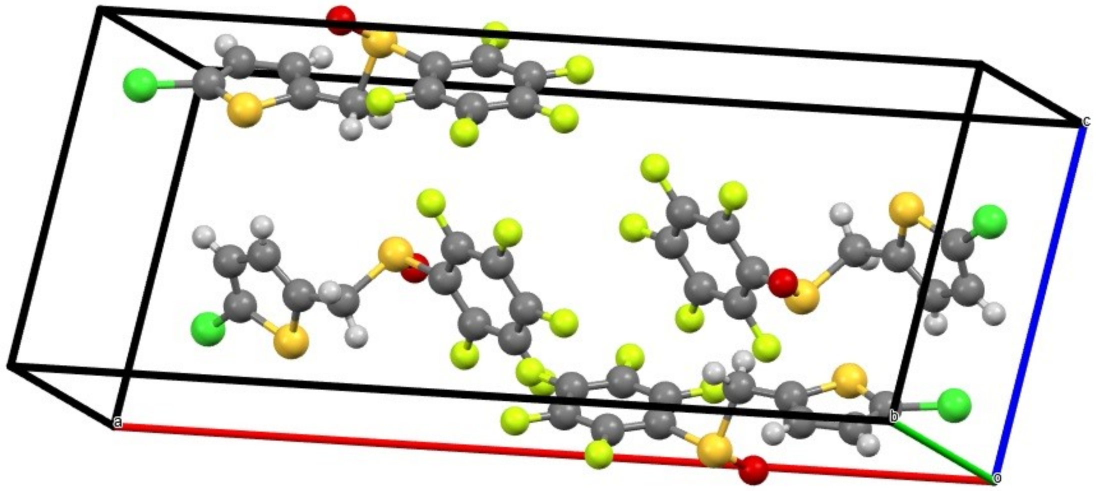

Figure S14. Packing plot of *rac*-2-chloro-5-((2, 3, 4, 5, 6-pentafluorophenylsulfinyl)methyl) thiophene **7**

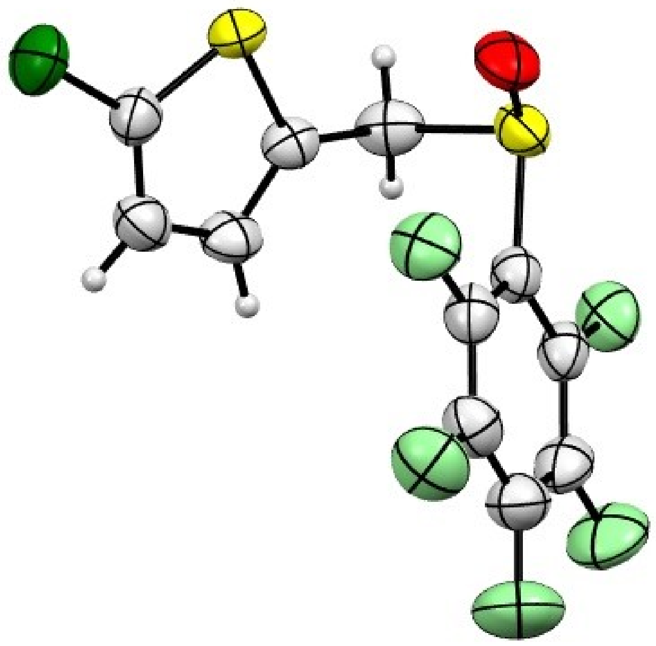

Figure S15. ORTEP plot (50% probability level) of (*R*)-2-chloro-5-((2, 3, 4, 5, 6-pentafluorophenylsulfinyl)methyl)thiophene **7**

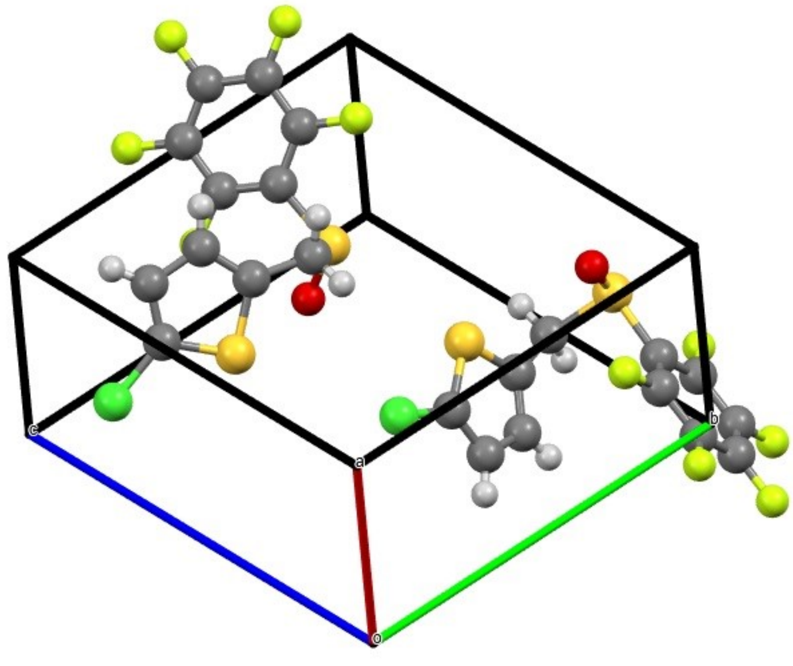

Figure S16. Packing plot of (*R*)-2-chloro-5-((2, 3, 4, 5, 6-pentafluorophenylsulfinyl)methyl) thiophene **7**

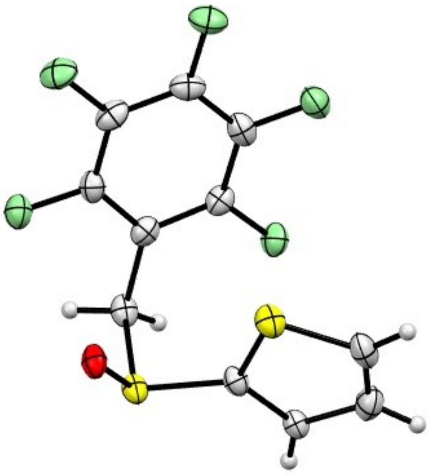

Figure S17. ORTEP plot (50% probability level) of *rac*-2-(2, 3, 4, 5, 6-pentafluorobenzyl)sulfinyl thiophene **8**.

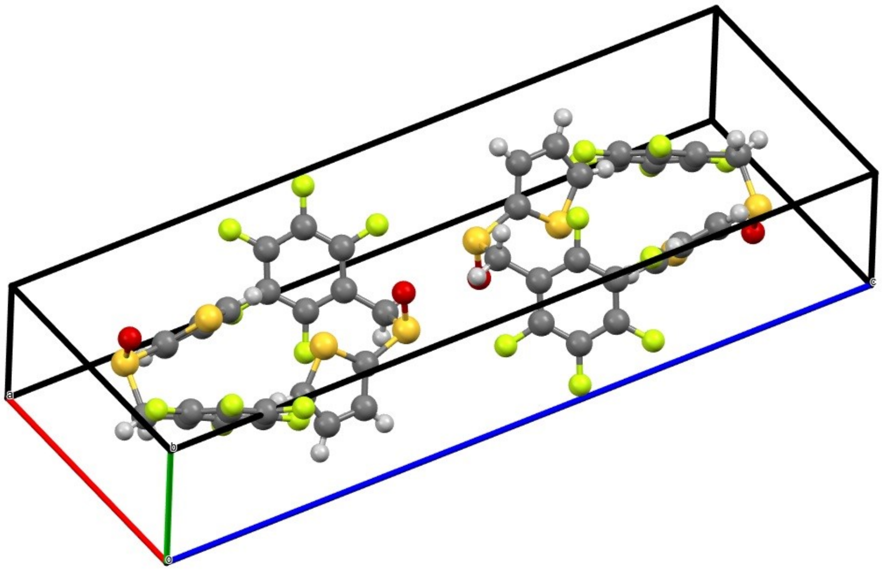

Figure S18. Packing plot of *rac*-2-(2, 3, 4, 5, 6-pentafluorobenzyl)sulfinyl thiophene **8**.

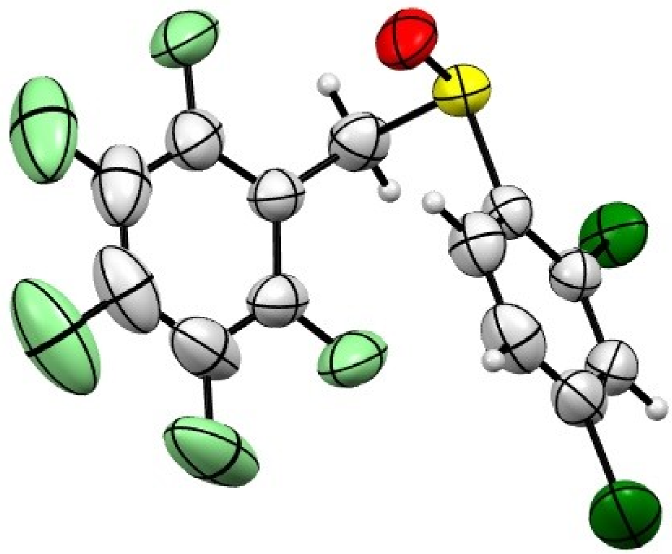

Figure S19. ORTEP plot (50% probability level) of *rac*-2,4-dichlorophenyl 2, 3, 4, 5, 6-pentafluorobenzyl sulfoxide **9**

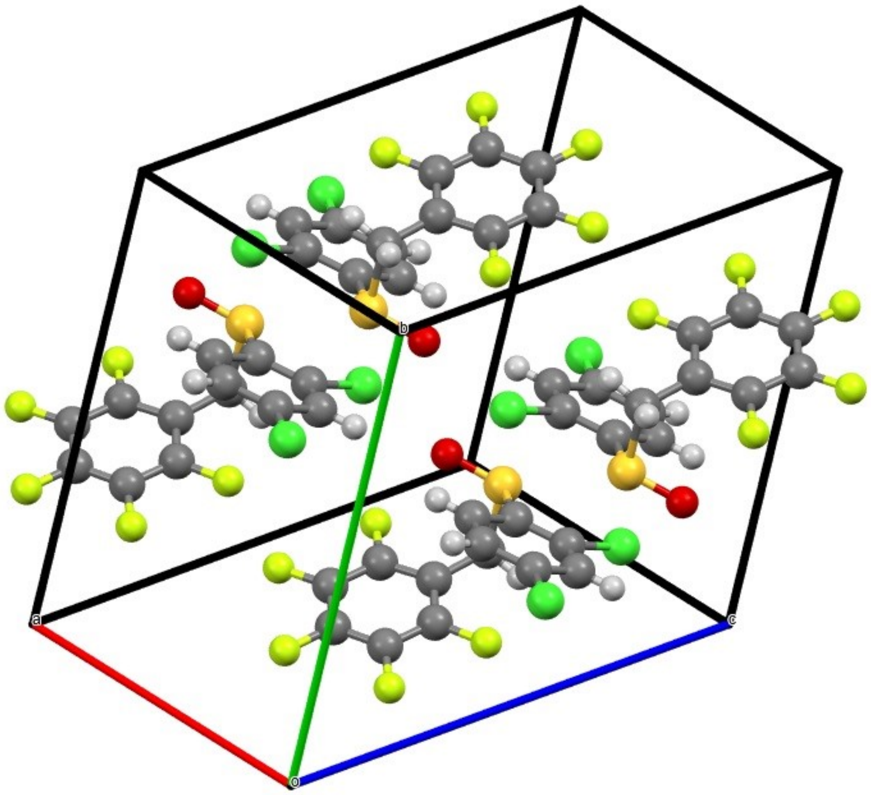

Figure S20. Packing plot of *rac*- 2,4-dichlorophenyl 2, 3, 4, 5, 6-pentafluorobenzyl sulfoxide **9**.

Outputs of pairwise lattice energy calculations (Crystal Explorer 21).

B3LYP/6-31G(d,p) calculated electronic, polarisation, dispersion and repulsion energies. The molecule interacting with the central molecule is identified by the distance R between the centroids of the molecules and the Symop (symmetry operator) connecting them. Further information on these output Tables can be obtained in the cited paper[24] and references therein. Molecules belonging to the "inner sphere" (see Text) are outlined with a yellow background. Molecules belonging to the "outer shell" (see Text) are outlined with a red background, if significant.

Table S11. *Crystal Explorer 21* estimate of lattice energies (kJ/mol) for *rac*-benzyl phenyl sulfoxide **1**

N	Symop	R	Electron Density	E_ele	E_pol	E_dis	E_rep	E_tot		
2	x, y, z	5,47	B3LYP/6-31G(d,p)	-18,7	-6,6	-31,4	25,7	-36,1	-36,1	
2	x+1/2, -y, z	4,84	B3LYP/6-31G(d,p)	-15,3	-5,2	-36,6	31	-32,8	-32,8	
2	x+1/2, -y, z	5,07	B3LYP/6-31G(d,p)	-7	-1,8	-38,1	24,6	-26,7	-26,7	-95,6
2	-x, -y, z+1/2	12,48	B3LYP/6-31G(d,p)	-0,9	-0,3	-6,6	0	-6,8	-6,8	
2	-x, -y, z+1/2	12,39	B3LYP/6-31G(d,p)	2,6	-0,4	-9	0	-5,4	-5,4	
2	-x+1/2, y, z+1/2	12,22	B3LYP/6-31G(d,p)	2,5	-0,2	-7,1	0	-3,7	-3,7	
2	x, y, z	10,94	B3LYP/6-31G(d,p)	-1	-0,1	-0,3	0	-1,4	-1,4	
2	-x+1/2, y, z+1/2	13,39	B3LYP/6-31G(d,p)	-0,2	-0,1	-0,9	0	-1,1	-1,1	
2	x, y, z	8,26	B3LYP/6-31G(d,p)	0,3	-0,1	-1,4	0	-1	-1	
2	-x+1/2, y, z+1/2	13,38	B3LYP/6-31G(d,p)	-0,2	0	-0,9	0	-1	-1	
2	x, y, z	9,9	B3LYP/6-31G(d,p)	-0,2	-0,1	-0,5	0	-0,7	-0,7	
2	-x+1/2, y, z+1/2	13,39	B3LYP/6-31G(d,p)	-0,1	0	-0,7	0	-0,7	-0,7	
2	-x, -y, z+1/2	13,86	B3LYP/6-31G(d,p)	-0,3	0	-0,3	0	-0,6	-0,6	
2	-x, -y, z+1/2	13,94	B3LYP/6-31G(d,p)	-0,2	0	-0,4	0	-0,6	-0,6	
2	-x, -y, z+1/2	14,53	B3LYP/6-31G(d,p)	-0,4	0	-0,2	0	-0,6	-0,6	
2	x+1/2, -y, z	9,37	B3LYP/6-31G(d,p)	0,5	-0,1	-1	0	-0,4	-0,4	
2	x+1/2, -y, z	12,64	B3LYP/6-31G(d,p)	-0,3	0	-0,1	0	-0,4	-0,4	
2	-x+1/2, y, z+1/2	14,45	B3LYP/6-31G(d,p)	-0,2	0	-0,3	0	-0,4	-0,4	
2	-x+1/2, y, z+1/2	16,4	B3LYP/6-31G(d,p)	-0,3	0	-0,1	0	-0,4	-0,4	
2	x+1/2, -y, z	12,73	B3LYP/6-31G(d,p)	-0,2	0	-0,1	0	-0,3	-0,3	
2	-x, -y, z+1/2	15,85	B3LYP/6-31G(d,p)	-0,2	0	-0,1	0	-0,3	-0,3	
2	-x, -y, z+1/2	14,76	B3LYP/6-31G(d,p)	0	0	-0,2	0	-0,2	-0,2	
2	-x+1/2, y, z+1/2	17,28	B3LYP/6-31G(d,p)	-0,2	0	0	0	-0,2	-0,2	
2	-x, -y, z+1/2	17,58	B3LYP/6-31G(d,p)	-0,1	0	0	0	-0,2	-0,2	
2	-x, -y, z+1/2	15,92	B3LYP/6-31G(d,p)	-0,1	0	-0,1	0	-0,1	-0,1	
2	-x, -y, z+1/2	16,01	B3LYP/6-31G(d,p)	0	0	-0,1	0	-0,1	-0,1	
2	-x+1/2, y, z+1/2	16,4	B3LYP/6-31G(d,p)	0	0	-0,1	0	-0,1	-0,1	
2	x, y, z	13,71	B3LYP/6-31G(d,p)	0,2	0	-0,1	0	0,1	0,1	
2	-x+1/2, y, z+1/2	14,45	B3LYP/6-31G(d,p)	0,3	0	-0,3	0	0,1	0,1	
2	-x+1/2, y, z+1/2	17,28	B3LYP/6-31G(d,p)	0,2	0	0	0	0,1	0,1	
2	-x+1/2, y, z+1/2	16	B3LYP/6-31G(d,p)	0,2	0	-0,1	0	0,2	0,2	
2	x, y, z	9,9	B3LYP/6-31G(d,p)	0,8	0	-0,6	0	0,3	0,3	
2	x+1/2, -y, z	9	B3LYP/6-31G(d,p)	2,2	-0,3	-1	0	1,2	1,2	
									-120,3	

Table S12. *Crystal Explorer 21* estimate of lattice energies (kJ/mol) for (*R*)-benzyl phenyl sulfoxide **1**

N	Symop	R	Electron Density	E_ele	E_pol	E_dis	E_rep	E_tot		
2	x, y, z	5,67	B3LYP/6-31G(d,p)	-18,8	-6,4	-29	25,5	-34,2	-34,2	
2	x+1/2, -y+1/2, -z	4,97	B3LYP/6-31G(d,p)	-6,4	-1,9	-38,5	21,9	-28,2	-28,2	-62,4
2	-x+1/2, -y, z+1/2	8,98	B3LYP/6-31G(d,p)	-4,3	-0,8	-13,5	8,2	-11,8	-11,8	
2	-x, y+1/2, -z+1/2	8,76	B3LYP/6-31G(d,p)	-5,8	-1,9	-7,9	5,4	-11	-11	
2	x+1/2, -y+1/2, -z	9,2	B3LYP/6-31G(d,p)	-1,9	-0,7	-15	7,6	-10,9	-10,9	
2	-x, y+1/2, -z+1/2	8,6	B3LYP/6-31G(d,p)	-1,9	-0,8	-15,1	10,3	-9,4	-9,4	-43,1
2	-x+1/2, -y, z+1/2	10,76	B3LYP/6-31G(d,p)	-1,3	-0,2	-0,9	0	-2,3	-2,3	
2	-x+1/2, -y, z+1/2	10,49	B3LYP/6-31G(d,p)	0,4	-0,1	-3,1	0,3	-2,1	-2,1	
2	x, y, z	11,34	B3LYP/6-31G(d,p)	-0,9	-0,1	-0,3	0	-1,3	-1,3	
2	x+1/2, -y+1/2, -z	12,21	B3LYP/6-31G(d,p)	-0,8	-0,1	-0,4	0	-1,3	-1,3	
2	x+1/2, -y+1/2, -z	9,44	B3LYP/6-31G(d,p)	-0,3	-0,1	-0,9	0	-1,1	-1,1	
2	-x+1/2, -y, z+1/2	12,28	B3LYP/6-31G(d,p)	-0,3	0	-0,6	0	-0,8	-0,8	
2	-x, y+1/2, -z+1/2	13,01	B3LYP/6-31G(d,p)	-0,4	0	-0,4	0	-0,7	-0,7	
2	x, y, z	11,4	B3LYP/6-31G(d,p)	0,2	0	-0,8	0	-0,5	-0,5	
2	-x, y+1/2, -z+1/2	11,64	B3LYP/6-31G(d,p)	0,1	0	-0,6	0	-0,4	-0,4	
2	-x, y+1/2, -z+1/2	15,37	B3LYP/6-31G(d,p)	-0,4	0	-0,1	0	-0,4	-0,4	
2	-x, y+1/2, -z+1/2	12,9	B3LYP/6-31G(d,p)	0	0	-0,4	0	-0,3	-0,3	
2	-x+1/2, -y, z+1/2	13,42	B3LYP/6-31G(d,p)	-0,1	0	-0,3	0	-0,3	-0,3	
2	-x+1/2, -y, z+1/2	13,63	B3LYP/6-31G(d,p)	-0,1	0	-0,2	0	-0,3	-0,3	
2	-x+1/2, -y, z+1/2	16,45	B3LYP/6-31G(d,p)	-0,3	0	-0,1	0	-0,3	-0,3	
2	x, y, z	12,74	B3LYP/6-31G(d,p)	0,2	0	-0,3	0	-0,1	-0,1	
2	x, y, z	16,09	B3LYP/6-31G(d,p)	-0,1	0	-0,1	0	-0,1	-0,1	
2	-x+1/2, -y, z+1/2	17,32	B3LYP/6-31G(d,p)	0	0	0	0	-0,1	-0,1	
2	-x+1/2, -y, z+1/2	14,66	B3LYP/6-31G(d,p)	0,1	0	-0,1	0	0	0	
2	x+1/2, -y+1/2, -z	20,68	B3LYP/6-31G(d,p)	0,1	0	0	0	0	0	
2	-x, y+1/2, -z+1/2	12	B3LYP/6-31G(d,p)	0,4	-0,1	-0,3	0	0,1	0,1	
2	-x+1/2, -y, z+1/2	14,28	B3LYP/6-31G(d,p)	0,2	0	-0,1	0	0,1	0,1	
2	x+1/2, -y+1/2, -z	15	B3LYP/6-31G(d,p)	0,1	0	-0,1	0	0,1	0,1	
2	-x, y+1/2, -z+1/2	15,1	B3LYP/6-31G(d,p)	0,1	0	-0,1	0	0,1	0,1	
2	x, y, z	20,62	B3LYP/6-31G(d,p)	0,1	0	0	0	0,1	0,1	
2	x, y, z	12,74	B3LYP/6-31G(d,p)	0,4	0	-0,3	0	0,2	0,2	
2	x+1/2, -y+1/2, -z	15,08	B3LYP/6-31G(d,p)	0,4	0	-0,1	0	0,3	0,3	
									-116,9	

Table S13. *Crystal Explorer 21* estimate of lattice energies (kJ/mol) for -*rac*-4-bromophenyl 2-methoxybenzyl sulfoxide **2**

N	Symop	R	Electron Density	E_ele	E_pol	E_dis	E_rep	E_tot		
2	x, y, z	7,3	B3LYP/6-31G(d,p)	-10,2	-4,4	-27,2	18	-26,6	-26,6	
1	-x, -y, -z	9,84	B3LYP/6-31G(d,p)	-25,9	-7	-46,6	32,6	-53	-26,5	
1	-x, -y, -z	6,96	B3LYP/6-31G(d,p)	-8,7	-0,9	-39,9	34,2	-23,6	-11,8	
1	-x, -y, -z	10,33	B3LYP/6-31G(d,p)	-0,1	-1,1	-35,3	14,4	-22,8	-11,4	
1	-x, -y, -z	6,92	B3LYP/6-31G(d,p)	-8,6	-5,2	-14,1	6,6	-21,1	-10,55	
1	-x, -y, -z	6,97	B3LYP/6-31G(d,p)	-5,6	-0,8	-31,1	21,7	-20,1	-10,05	
2	x, y, z	15,03	B3LYP/6-31G(d,p)	-5,5	-0,1	-4,8	0	-10	-10	
1	-x, -y, -z	6,11	B3LYP/6-31G(d,p)	-3	-0,6	-23,1	13,4	-15,5	-7,75	
1	-x, -y, -z	8,8	B3LYP/6-31G(d,p)	-1,9	-1,8	-20,5	11,4	-14,1	-7,05	
2	x, y, z	12,36	B3LYP/6-31G(d,p)	1,5	-0,5	-7,1	0	-4,9	-4,9	
1	-x, -y, -z	14,9	B3LYP/6-31G(d,p)	-0,9	-0,2	-8,7	0	-8,6	-4,3	
2	x, y, z	7,65	B3LYP/6-31G(d,p)	0,3	-0,4	-2,9	0	-2,4	-2,4	-102,7
1	-x, -y, -z	14,93	B3LYP/6-31G(d,p)	-1,1	-0,1	-1	0	-2,1	-1,05	
2	x, y, z	10,06	B3LYP/6-31G(d,p)	0,3	-0,1	-1,4	0	-0,9	-0,9	
2	x, y, z	13,64	B3LYP/6-31G(d,p)	-0,6	0	-0,3	0	-0,9	-0,9	
1	-x, -y, -z	9,9	B3LYP/6-31G(d,p)	0,3	-0,1	-2,1	0	-1,6	-0,8	
2	x, y, z	13,82	B3LYP/6-31G(d,p)	-0,3	0	-0,5	0	-0,8	-0,8	
1	-x, -y, -z	13,19	B3LYP/6-31G(d,p)	-1,2	0	-0,2	0	-1,5	-0,75	
2	x, y, z	11,07	B3LYP/6-31G(d,p)	-0,2	-0,1	-0,5	0	-0,7	-0,7	
1	-x, -y, -z	12,91	B3LYP/6-31G(d,p)	-0,9	0	-0,5	0	-1,4	-0,7	
1	-x, -y, -z	14,28	B3LYP/6-31G(d,p)	-1,2	0	-0,2	0	-1,4	-0,7	
1	-x, -y, -z	12,48	B3LYP/6-31G(d,p)	-0,6	0	-0,7	0	-1,3	-0,65	
2	x, y, z	15,22	B3LYP/6-31G(d,p)	-0,3	0	-0,2	0	-0,5	-0,5	
1	-x, -y, -z	11,53	B3LYP/6-31G(d,p)	-0,2	0	-0,6	0	-0,8	-0,4	
2	x, y, z	16,58	B3LYP/6-31G(d,p)	-0,1	0	-0,2	0	-0,3	-0,3	
2	x, y, z	17,15	B3LYP/6-31G(d,p)	0	0	-0,2	0	-0,2	-0,2	
2	x, y, z	20,15	B3LYP/6-31G(d,p)	-0,1	0	-0,1	0	-0,2	-0,2	
1	-x, -y, -z	17,46	B3LYP/6-31G(d,p)	-0,2	0	-0,1	0	-0,3	-0,15	
1	-x, -y, -z	12	B3LYP/6-31G(d,p)	0	0	-0,2	0	-0,2	-0,1	
2	x, y, z	14,61	B3LYP/6-31G(d,p)	0	0	-0,2	0	-0,1	-0,1	
2	x, y, z	14,62	B3LYP/6-31G(d,p)	0	0	-0,1	0	-0,1	-0,1	
1	-x, -y, -z	21,07	B3LYP/6-31G(d,p)	-0,2	0	-0,1	0	-0,2	-0,1	
1	-x, -y, -z	18,39	B3LYP/6-31G(d,p)	-0,1	0	-0,1	0	-0,1	-0,05	
1	-x, -y, -z	16,54	B3LYP/6-31G(d,p)	0	0	-0,1	0	0	0	
1	-x, -y, -z	14	B3LYP/6-31G(d,p)	0,2	0	-0,1	0	0,1	0,05	
1	-x, -y, -z	12,84	B3LYP/6-31G(d,p)	0,4	0	-0,3	0	0,2	0,1	
1	-x, -y, -z	21,24	B3LYP/6-31G(d,p)	0,2	0	-0,1	0	0,2	0,1	
2	x, y, z	15,83	B3LYP/6-31G(d,p)	0,3	0	-0,1	0	0,2	0,2	
1	-x, -y, -z	12,41	B3LYP/6-31G(d,p)	0,7	-0,1	-0,3	0	0,5	0,25	
1	-x, -y, -z	13,24	B3LYP/6-31G(d,p)	0,6	0	-0,1	0	0,5	0,25	
1	-x, -y, -z	20,29	B3LYP/6-31G(d,p)	0,6	0	-0,1	0	0,5	0,25	
									-142,25	

Table S14. *Crystal Explorer 21* estimate of lattice energies (kJ/mol) for (*R*)-4-bromophenyl 2-methoxybenzyl sulfoxide **2**

N	Symop	R	Electron Density	E_ele	E_pol	E_dis	E_rep	E_tot		
2	x, y, z	7,78	B3LYP/6-31G(d,p)	-5,7	-1,5	-44,2	26,5	-29,3	-29,3	
2	-x, y+1/2, -z	9,18	B3LYP/6-31G(d,p)	-10,2	-2	-25,6	16,4	-24,4	-24,4	
2	x, y, z	7,72	B3LYP/6-31G(d,p)	-15,4	-4,4	-19	23,2	-21,7	-21,7	
2	-x, y+1/2, -z	7,88	B3LYP/6-31G(d,p)	-13	-0,7	-18,8	24,9	-15,2	-15,2	
2	-x, y+1/2, -z	6,99	B3LYP/6-31G(d,p)	-2,5	-1	-16	5,6	-13,8	-13,8	
2	-x, y+1/2, -z	8,31	B3LYP/6-31G(d,p)	-2,2	-1,1	-17,1	9,3	-12,3	-12,3	
2	x, y, z	12,18	B3LYP/6-31G(d,p)	0	-0,3	-7,7	0	-6,9	-6,9	
2	x, y, z	9,58	B3LYP/6-31G(d,p)	-1,7	-0,3	-1,7	0	-3,5	-3,5	-120,2
2	-x, y+1/2, -z	14,78	B3LYP/6-31G(d,p)	-0,7	0	-0,5	0	-1,2	-1,2	
2	-x, y+1/2, -z	15,51	B3LYP/6-31G(d,p)	-0,4	0	-0,9	0	-1,2	-1,2	
2	x, y, z	14,03	B3LYP/6-31G(d,p)	-0,1	0	-0,7	0	-0,7	-0,7	
2	x, y, z	15,1	B3LYP/6-31G(d,p)	-0,4	0	-0,1	0	-0,6	-0,6	
2	-x, y+1/2, -z	13,95	B3LYP/6-31G(d,p)	-0,3	0	-0,2	0	-0,5	-0,5	
2	-x, y+1/2, -z	14,04	B3LYP/6-31G(d,p)	0,2	-0,1	-0,6	0	-0,4	-0,4	
2	x, y, z	16,88	B3LYP/6-31G(d,p)	-0,1	0	-0,2	0	-0,3	-0,3	
2	x, y, z	19,45	B3LYP/6-31G(d,p)	-0,2	0	-0,1	0	-0,3	-0,3	
2	-x, y+1/2, -z	20,08	B3LYP/6-31G(d,p)	-0,2	0	-0,1	0	-0,3	-0,3	
2	-x, y+1/2, -z	11,59	B3LYP/6-31G(d,p)	0,3	0	-0,6	0	-0,2	-0,2	
2	x, y, z	15,65	B3LYP/6-31G(d,p)	-0,1	0	-0,1	0	-0,2	-0,2	
2	-x, y+1/2, -z	13,16	B3LYP/6-31G(d,p)	0,1	0	-0,2	0	-0,1	-0,1	
2	x, y, z	15,44	B3LYP/6-31G(d,p)	0	0	-0,1	0	-0,1	-0,1	
2	x, y, z	18,86	B3LYP/6-31G(d,p)	0,1	0	-0,1	0	0	0	
2	x, y, z	18,93	B3LYP/6-31G(d,p)	0	0	-0,1	0	0	0	
2	x, y, z	11,68	B3LYP/6-31G(d,p)	0,4	0	-0,4	0	0,1	0,1	
2	-x, y+1/2, -z	13,27	B3LYP/6-31G(d,p)	0,7	0	-0,7	0	0,1	0,1	
2	x, y, z	14	B3LYP/6-31G(d,p)	0,2	0	-0,1	0	0,1	0,1	
2	x, y, z	15,55	B3LYP/6-31G(d,p)	0,4	0	-0,3	0	0,2	0,2	
2	-x, y+1/2, -z	14,56	B3LYP/6-31G(d,p)	0,4	0	-0,1	0	0,3	0,3	
2	x, y, z	14	B3LYP/6-31G(d,p)	0,6	0	-0,2	0	0,4	0,4	
2	-x, y+1/2, -z	12,44	B3LYP/6-31G(d,p)	0,9	-0,1	-0,4	0	0,5	0,5	
2	-x, y+1/2, -z	16,52	B3LYP/6-31G(d,p)	0,5	0	-0,1	0	0,5	0,5	
									-131	

Table S15. *Crystal Explorer 21* estimate of lattice energies (kJ/mol) for *rac*-4-bromophenyl 2-nitrobenzyl sulfoxide **3**

N	Symop	R	Electron Density	E_ele	E_pol	E_dis	E_rep	E_tot		
2	x, y, z	7,6	B3LYP/6-31G(d,p)	-7,5	-1,4	-36,7	17,8	-29,9	-29,9	
2	x+1/2, -y+1/2, z+1/2	7,12	B3LYP/6-31G(d,p)	-16,5	-5,4	-18	20,4	-24,6	-24,6	
1	-x, -y, -z	10,08	B3LYP/6-31G(d,p)	-16	-3,6	-37,9	25,5	-36,8	-18,4	
2	x+1/2, -y+1/2, z+1/2	8,28	B3LYP/6-31G(d,p)	-5,9	-1,4	-8,6	3,1	-12,7	-12,7	
1	-x, -y, -z	6,68	B3LYP/6-31G(d,p)	-4,5	-1,2	-30	18,6	-20,3	-10,15	
2	-x+1/2, y+1/2, -z+1/2	8,53	B3LYP/6-31G(d,p)	-0,7	-1,3	-12,1	4,9	-9,1	-9,1	
2	-x+1/2, y+1/2, -z+1/2	9	B3LYP/6-31G(d,p)	-4,5	-0,6	-10,4	8,8	-8,8	-8,8	-113,7
1	-x, -y, -z	11,33	B3LYP/6-31G(d,p)	-4,4	-0,3	-10,5	9,1	-8,4	-4,2	
2	x+1/2, -y+1/2, z+1/2	12,18	B3LYP/6-31G(d,p)	2,6	-0,5	-6,9	0	-3,6	-3,6	
2	-x+1/2, y+1/2, -z+1/2	11,47	B3LYP/6-31G(d,p)	-0,8	-0,1	-0,8	0	-1,6	-1,6	
1	-x, -y, -z	15,78	B3LYP/6-31G(d,p)	2	-0,6	-4,9	0	-2,6	-1,3	
1	-x, -y, -z	8,36	B3LYP/6-31G(d,p)	0,9	-0,2	-2,5	0	-1,4	-0,7	
2	-x+1/2, y+1/2, -z+1/2	13,42	B3LYP/6-31G(d,p)	-0,1	0	-0,6	0	-0,7	-0,7	
1	-x, -y, -z	8,75	B3LYP/6-31G(d,p)	0,6	-0,1	-1,9	0	-1,1	-0,55	
2	x, y, z	14,12	B3LYP/6-31G(d,p)	-0,4	0	-0,2	0	-0,5	-0,5	
2	-x+1/2, y+1/2, -z+1/2	14,3	B3LYP/6-31G(d,p)	-0,2	0	-0,3	0	-0,5	-0,5	
1	-x, -y, -z	13,91	B3LYP/6-31G(d,p)	-0,2	0	-0,3	0	-0,5	-0,25	
2	-x+1/2, y+1/2, -z+1/2	13,24	B3LYP/6-31G(d,p)	0	0	-0,2	0	-0,2	-0,2	
2	-x+1/2, y+1/2, -z+1/2	14,26	B3LYP/6-31G(d,p)	0,4	-0,1	-0,5	0	-0,1	-0,1	
2	x+1/2, -y+1/2, z+1/2	14,56	B3LYP/6-31G(d,p)	0,1	0	-0,1	0	-0,1	-0,1	
2	x, y, z	18,36	B3LYP/6-31G(d,p)	-0,1	0	0	0	-0,1	-0,1	
2	x+1/2, -y+1/2, z+1/2	19,02	B3LYP/6-31G(d,p)	-0,1	0	-0,1	0	-0,1	-0,1	
1	-x, -y, -z	20,78	B3LYP/6-31G(d,p)	-0,1	0	0	0	-0,1	-0,05	
1	-x, -y, -z	12,06	B3LYP/6-31G(d,p)	0,2	0	-0,2	0	0	0	
1	-x, -y, -z	13,06	B3LYP/6-31G(d,p)	0,2	0	-0,2	0	0	0	
2	x, y, z	20,43	B3LYP/6-31G(d,p)	0,1	0	-0,1	0	0	0	
1	-x, -y, -z	18,1	B3LYP/6-31G(d,p)	0,1	0	-0,1	0	0,1	0,05	
1	-x, -y, -z	22,62	B3LYP/6-31G(d,p)	0,1	0	0	0	0,1	0,05	
2	x, y, z	15,2	B3LYP/6-31G(d,p)	0,3	0	-0,2	0	0,1	0,1	
2	x+1/2, -y+1/2, z+1/2	16,2	B3LYP/6-31G(d,p)	0,2	0	-0,1	0	0,1	0,1	
1	-x, -y, -z	15,43	B3LYP/6-31G(d,p)	0,7	0	-0,2	0	0,5	0,25	
2	x, y, z	13,66	B3LYP/6-31G(d,p)	0,5	0	-0,2	0	0,3	0,3	
2	x, y, z	13,32	B3LYP/6-31G(d,p)	0,5	0	-0,2	0	0,4	0,4	
2	x, y, z	15,63	B3LYP/6-31G(d,p)	0,7	0	-0,3	0	0,4	0,4	
2	x+1/2, -y+1/2, z+1/2	14,2	B3LYP/6-31G(d,p)	0,7	0	-0,3	0	0,5	0,5	
2	-x+1/2, y+1/2, -z+1/2	19,76	B3LYP/6-31G(d,p)	0,5	0	-0,1	0	0,5	0,5	
									-125,6	

Table S16. *Crystal Explorer 21* estimate of lattice energies (kJ/mol) for (*R*)-4-bromophenyl 2-nitrobenzyl sulfoxide **3**

N	Symop	R	Electron Density	E_ele	E_pol	E_dis	E_rep	E_tot		
2	x, y, z	5,58	B3LYP/6-31G(d,p)	-21,2	-7	-35,2	31,6	-38,6	-38,6	
2	x+1/2, -y+1/2, -z	6,12	B3LYP/6-31G(d,p)	-12,4	-4,3	-40,2	28	-34,1	-34,1	
2	-x+1/2, -y, z+1/2	9,29	B3LYP/6-31G(d,p)	-6,4	-0,5	-14,2	11	-12,7	-12,7	
2	x+1/2, -y+1/2, -z	9,34	B3LYP/6-31G(d,p)	-4,8	-0,4	-17	13,5	-11,9	-11,9	
2	-x, y+1/2, -z+1/2	9,92	B3LYP/6-31G(d,p)	-4,8	-0,8	-10,5	9,4	-9,1	-9,1	
2	x+1/2, -y+1/2, -z	9,98	B3LYP/6-31G(d,p)	-1,5	-0,1	-1,1	0	-2,7	-2,7	
2	-x, y+1/2, -z+1/2	10,08	B3LYP/6-31G(d,p)	-1,6	-1,2	-10,1	3,5	-9,2	-9,2	-79,7
2	-x+1/2, -y, z+1/2	10,68	B3LYP/6-31G(d,p)	-0,1	0	-1,2	0	-1,2	-1,2	
2	-x+1/2, -y, z+1/2	10,98	B3LYP/6-31G(d,p)	0,5	-0,3	-3,1	2,1	-1,1	-1,1	
2	x, y, z	11,15	B3LYP/6-31G(d,p)	0,4	0	-0,5	0	0	0	
2	x+1/2, -y+1/2, -z	12,22	B3LYP/6-31G(d,p)	0,4	0	-0,5	0	0	0	
2	-x, y+1/2, -z+1/2	12,54	B3LYP/6-31G(d,p)	-0,3	-0,1	-0,6	0	-0,9	-0,9	
2	-x, y+1/2, -z+1/2	12,92	B3LYP/6-31G(d,p)	-0,1	0	-0,4	0	-0,5	-0,5	
2	-x, y+1/2, -z+1/2	13,49	B3LYP/6-31G(d,p)	-0,6	0	-0,3	0	-0,9	-0,9	
2	x, y, z	13,55	B3LYP/6-31G(d,p)	-0,3	0	-0,7	0	-1	-1	
2	-x, y+1/2, -z+1/2	13,61	B3LYP/6-31G(d,p)	-1,1	0	-0,3	0	-1,4	-1,4	
2	-x+1/2, -y, z+1/2	14,29	B3LYP/6-31G(d,p)	0	0	-0,1	0	-0,2	-0,2	
2	x, y, z	14,65	B3LYP/6-31G(d,p)	-0,1	0	-0,4	0	-0,5	-0,5	
2	x, y, z	14,65	B3LYP/6-31G(d,p)	-0,2	0	-0,2	0	-0,4	-0,4	
2	-x+1/2, -y, z+1/2	14,73	B3LYP/6-31G(d,p)	0,1	0	-0,1	0	0	0	
2	-x+1/2, -y, z+1/2	14,84	B3LYP/6-31G(d,p)	0	0	-0,4	0	-0,4	-0,4	
2	-x, y+1/2, -z+1/2	15,52	B3LYP/6-31G(d,p)	0	0	-0,1	0	-0,1	-0,1	
2	-x+1/2, -y, z+1/2	15,75	B3LYP/6-31G(d,p)	-0,4	0	-0,2	0	-0,7	-0,7	
2	-x+1/2, -y, z+1/2	15,95	B3LYP/6-31G(d,p)	-0,2	0	-0,1	0	-0,4	-0,4	
2	x+1/2, -y+1/2, -z	16,88	B3LYP/6-31G(d,p)	-0,5	0	-0,1	0	-0,7	-0,7	
2	x, y, z	17,55	B3LYP/6-31G(d,p)	-0,1	0	-0,1	0	-0,1	-0,1	
2	x+1/2, -y+1/2, -z	18,84	B3LYP/6-31G(d,p)	0,3	0	-0,1	0	0,3	0,3	
									-128,5	

Table S17. *Crystal Explorer 21* estimate of lattice energies (kJ/mol) for *rac*-4-bromophenyl 4-nitrobenzyl sulfoxide **4**

N	Symop	R	Electron Density	E_ele	E_pol	E_dis	E_rep	E_tot		
2	x, y, z	5,58	B3LYP/6-31G(d,p)	-16,5	-5,4	-38,4	30,6	-36	-36	
2	-x, y+1/2, -z+1/2	8,57	B3LYP/6-31G(d,p)	-7,2	-1,5	-25,8	17,1	-20,7	-20,7	
2	-x, y+1/2, -z+1/2	8,58	B3LYP/6-31G(d,p)	-10,1	-1,7	-26,2	25,1	-19,3	-19,3	
1	-x, -y, -z	7,66	B3LYP/6-31G(d,p)	-20,6	-5,7	-21,4	19,5	-32,5	-16,25	
1	-x, -y, -z	6,97	B3LYP/6-31G(d,p)	-17,7	-4	-23,1	17,6	-30,9	-15,45	
1	-x, -y, -z	10,06	B3LYP/6-31G(d,p)	-11,1	-3,3	-15,1	10,2	-21	-10,5	
2	x, -y+1/2, z+1/2	9,82	B3LYP/6-31G(d,p)	-9,8	-1,4	-7,5	18,5	-6,5	-6,5	
2	x, -y+1/2, z+1/2	8,69	B3LYP/6-31G(d,p)	-5,9	-0,9	-12,9	19,8	-5,9	-5,9	-94,6
2	x, -y+1/2, z+1/2	13,71	B3LYP/6-31G(d,p)	-1	-0,2	-0,7	0	-1,8	-1,8	
1	-x, -y, -z	10,04	B3LYP/6-31G(d,p)	-2,4	-0,1	-0,9	0	-3,4	-1,7	
1	-x, -y, -z	12,37	B3LYP/6-31G(d,p)	-2,1	-0,1	-0,9	0	-3,1	-1,55	
2	x, y, z	11,16	B3LYP/6-31G(d,p)	-0,7	-0,1	-0,5	0	-1,2	-1,2	
1	-x, -y, -z	13,66	B3LYP/6-31G(d,p)	-1,7	0	-0,5	0	-2,2	-1,1	
2	x, -y+1/2, z+1/2	15,14	B3LYP/6-31G(d,p)	-0,8	-0,1	-0,2	0	-1,1	-1,1	
2	-x, y+1/2, -z+1/2	11,66	B3LYP/6-31G(d,p)	0	-0,1	-0,7	0	-0,8	-0,8	
2	x, -y+1/2, z+1/2	10,81	B3LYP/6-31G(d,p)	0,1	-0,1	-0,8	0	-0,7	-0,7	
2	x, y, z	15,03	B3LYP/6-31G(d,p)	-0,4	0	-0,1	0	-0,6	-0,6	
2	x, -y+1/2, z+1/2	13,4	B3LYP/6-31G(d,p)	-0,3	0	-0,3	0	-0,5	-0,5	
1	-x, -y, -z	15,45	B3LYP/6-31G(d,p)	-0,8	0	-0,2	0	-1	-0,5	
2	x, -y+1/2, z+1/2	14,85	B3LYP/6-31G(d,p)	-0,3	0	-0,1	0	-0,4	-0,4	
2	-x, y+1/2, -z+1/2	15,38	B3LYP/6-31G(d,p)	-0,3	0	-0,1	0	-0,4	-0,4	
1	-x, -y, -z	14,03	B3LYP/6-31G(d,p)	-0,4	0	-0,2	0	-0,6	-0,3	
1	-x, -y, -z	11,45	B3LYP/6-31G(d,p)	0,1	-0,1	-0,6	0	-0,5	-0,25	
1	-x, -y, -z	16,34	B3LYP/6-31G(d,p)	-0,2	0	-0,1	0	-0,3	-0,15	
1	-x, -y, -z	15,8	B3LYP/6-31G(d,p)	-0,1	0	-0,1	0	-0,2	-0,1	
2	x, y, z	16,78	B3LYP/6-31G(d,p)	0,1	0	-0,1	0	0	0	
2	-x, y+1/2, -z+1/2	11,64	B3LYP/6-31G(d,p)	1	-0,1	-0,8	0	0,3	0,3	
2	x, y, z	15,83	B3LYP/6-31G(d,p)	0,4	0	-0,1	0	0,3	0,3	
2	-x, y+1/2, -z+1/2	16,03	B3LYP/6-31G(d,p)	0,4	0	-0,1	0	0,3	0,3	
2	x, y, z	15,38	B3LYP/6-31G(d,p)	0,5	0	-0,1	0	0,4	0,4	
2	x, -y+1/2, z+1/2	14,45	B3LYP/6-31G(d,p)	0,9	0	-0,3	0	0,7	0,7	
1	-x, -y, -z	10,56	B3LYP/6-31G(d,p)	5,4	-0,9	-3,9	0,2	1,7	0,85	
1	-x, -y, -z	13,57	B3LYP/6-31G(d,p)	2	-0,1	-0,2	0	1,8	0,9	
									-140,0	

Table S18. *Crystal Explorer 21* estimate of lattice energies (kJ/mol) for (*R*)-4-bromophenyl 4-nitrobenzyl sulfoxide **4**

A	B	C	D	E	F	G	H	I	J	K
N	Symop	R	Electron Density	E_ele	E_pol	E_dis	E_rep	E_tot		
2	x, y, z	5,65	B3LYP/6-31G(d,p)	-16,2	-6,2	-37,2	28,2	-36,6	-36,6	
2	x+1/2, -y+1/2, -z	7,4	B3LYP/6-31G(d,p)	-11,5	-3,8	-22,4	13,6	-26	-26	
2	x+1/2, -y+1/2, -z	7,89	B3LYP/6-31G(d,p)	-6,3	-1,2	-25,3	19,5	-17,5	-17,5	
2	-x, y+1/2, -z+1/2	9,35	B3LYP/6-31G(d,p)	-1,3	-1,5	-17,8	6,6	-13,8	-13,8	
2	-x+1/2, -y, z+1/2	9,37	B3LYP/6-31G(d,p)	-2,2	-0,7	-9,9	11,2	-4,6	-4,6	
2	-x+1/2, -y, z+1/2	9,57	B3LYP/6-31G(d,p)	-4,8	-0,6	-12,3	11,3	-9,3	-9,3	-71,2
2	-x, y+1/2, -z+1/2	10,74	B3LYP/6-31G(d,p)	-4,4	-1,1	-6,2	2,2	-9,5	-9,5	
2	x+1/2, -y+1/2, -z	10,89	B3LYP/6-31G(d,p)	-0,9	-0,1	-0,7	0	-1,6	-1,6	
2	-x, y+1/2, -z+1/2	11,09	B3LYP/6-31G(d,p)	0,9	-0,3	-1,2	0	-0,2	-0,2	
2	x+1/2, -y+1/2, -z	11,22	B3LYP/6-31G(d,p)	0,1	-0,1	-0,7	0	-0,6	-0,6	
2	x, y, z	11,29	B3LYP/6-31G(d,p)	-0,7	-0,1	-0,4	0	-1,2	-1,2	
2	-x+1/2, -y, z+1/2	12,16	B3LYP/6-31G(d,p)	-0,6	0	-0,5	0	-1	-1	
2	-x+1/2, -y, z+1/2	12,62	B3LYP/6-31G(d,p)	0,2	-0,1	-0,4	0	-0,2	-0,2	
2	-x, y+1/2, -z+1/2	13,3	B3LYP/6-31G(d,p)	0,1	0	-0,3	0	-0,1	-0,1	
2	x, y, z	13,31	B3LYP/6-31G(d,p)	-0,3	-0,1	-0,5	0	-0,8	-0,8	
2	-x, y+1/2, -z+1/2	14,32	B3LYP/6-31G(d,p)	0,3	0	-0,1	0	0,2	0,2	
2	-x, y+1/2, -z+1/2	14,4	B3LYP/6-31G(d,p)	-0,2	0	-0,2	0	-0,4	-0,4	
2	x, y, z	14,46	B3LYP/6-31G(d,p)	-1	-0,1	-0,3	0	-1,4	-1,4	
2	x, y, z	14,46	B3LYP/6-31G(d,p)	0,7	0	-0,2	0	0,6	0,6	
2	-x, y+1/2, -z+1/2	14,58	B3LYP/6-31G(d,p)	-0,1	0	-0,2	0	-0,2	-0,2	
2	-x, y+1/2, -z+1/2	14,92	B3LYP/6-31G(d,p)	0,8	0	-0,1	0	0,8	0,8	
2	-x+1/2, -y, z+1/2	16,05	B3LYP/6-31G(d,p)	-0,2	0	-0,1	0	-0,4	-0,4	
2	-x+1/2, -y, z+1/2	16,17	B3LYP/6-31G(d,p)	-0,3	0	-0,1	0	-0,4	-0,4	
2	x+1/2, -y+1/2, -z	17,07	B3LYP/6-31G(d,p)	0,1	0	-0,1	0	0,1	0,1	
2	x+1/2, -y+1/2, -z	17,29	B3LYP/6-31G(d,p)	0,7	0	-0,1	0	0,7	0,7	
2	x, y, z	17,46	B3LYP/6-31G(d,p)	-0,4	0	-0,1	0	-0,5	-0,5	
2	-x+1/2, -y, z+1/2	17,82	B3LYP/6-31G(d,p)	0	0	0	0	-0,1	-0,1	
									-124	

Table S19. *Crystal Explorer 21* estimate of lattice energies (kJ/mol) for *rac*-4-bromophenyl 3-chlorobenzyl sulfoxide **5**

N	Symop	R	Electron Density	E_ele	E_pol	E_dis	E_rep	E_tot		
2	x, -y+1/2, z+1/2	5,42	B3LYP/6-31G(d,p)	-19,1	-6,6	-42,8	35,3	-40,5	-40,5	
2	-x, y+1/2, -z+1/2	5,35	B3LYP/6-31G(d,p)	-6,7	-2,1	-30,6	19,1	-23,5	-23,5	
1	-x, -y, -z	5,44	B3LYP/6-31G(d,p)	-24,1	-5,9	-28,5	25,9	-38,6	-19,3	
1	-x, -y, -z	4,35	B3LYP/6-31G(d,p)	-3,7	-2,1	-56,5	32,9	-34,3	-17,15	-100,45
2	-x, y+1/2, -z+1/2	15,85	B3LYP/6-31G(d,p)	-4,4	-0,1	-1,5	0	-6	-6	
2	x, y, z	14,77	B3LYP/6-31G(d,p)	1,1	-0,3	-7,2	0	-5,3	-5,3	
2	x, y, z	8,78	B3LYP/6-31G(d,p)	-2,8	-0,3	-1,5	0	-4,5	-4,5	
1	-x, -y, -z	14,44	B3LYP/6-31G(d,p)	2,5	-0,5	-11,8	0	-8,1	-4,05	
2	x, -y+1/2, z+1/2	14,64	B3LYP/6-31G(d,p)	1,5	-0,1	-3,4	0	-1,4	-1,4	
1	-x, -y, -z	14,49	B3LYP/6-31G(d,p)	-1,1	0	-1,7	0	-2,6	-1,3	
1	-x, -y, -z	9,86	B3LYP/6-31G(d,p)	-1,3	-0,1	-0,7	0	-2,1	-1,05	
2	x, -y+1/2, z+1/2	13,55	B3LYP/6-31G(d,p)	-0,8	0	-0,1	0	-1	-1	
2	-x, y+1/2, -z+1/2	15,56	B3LYP/6-31G(d,p)	-0,3	0	-0,5	0	-0,7	-0,7	
2	x, -y+1/2, z+1/2	8,66	B3LYP/6-31G(d,p)	0,8	-0,1	-1,5	0	-0,6	-0,6	
1	-x, -y, -z	9,3	B3LYP/6-31G(d,p)	0,5	-0,2	-1,7	0	-1,1	-0,55	
1	-x, -y, -z	16,89	B3LYP/6-31G(d,p)	-0,6	-0,1	-0,5	0	-1,1	-0,55	
2	-x, y+1/2, -z+1/2	10,79	B3LYP/6-31G(d,p)	0	-0,1	-0,4	0	-0,4	-0,4	
2	x, y, z	15,13	B3LYP/6-31G(d,p)	0,1	0	-0,6	0	-0,4	-0,4	
2	x, -y+1/2, z+1/2	16,12	B3LYP/6-31G(d,p)	-0,1	0	-0,3	0	-0,4	-0,4	
1	-x, -y, -z	16,61	B3LYP/6-31G(d,p)	-0,4	0	-0,3	0	-0,7	-0,35	
2	x, y, z	13,79	B3LYP/6-31G(d,p)	-0,2	0	-0,1	0	-0,3	-0,3	
2	-x, y+1/2, -z+1/2	16,24	B3LYP/6-31G(d,p)	-0,2	0	-0,1	0	-0,3	-0,3	
2	-x, y+1/2, -z+1/2	15,86	B3LYP/6-31G(d,p)	0,3	0	-0,5	0	-0,2	-0,2	
1	-x, -y, -z	16,3	B3LYP/6-31G(d,p)	0	0	-0,3	0	-0,3	-0,15	
2	x, -y+1/2, z+1/2	16,75	B3LYP/6-31G(d,p)	0,1	0	-0,2	0	-0,1	-0,1	
1	-x, -y, -z	16,91	B3LYP/6-31G(d,p)	0	0	-0,1	0	-0,2	-0,1	
2	x, -y+1/2, z+1/2	18,06	B3LYP/6-31G(d,p)	0	0	-0,1	0	-0,1	-0,1	
1	-x, -y, -z	16,66	B3LYP/6-31G(d,p)	0	0	-0,1	0	-0,1	-0,05	
1	-x, -y, -z	18,25	B3LYP/6-31G(d,p)	0	0	-0,1	0	-0,1	-0,05	
2	x, -y+1/2, z+1/2	17,39	B3LYP/6-31G(d,p)	0	0	-0,1	0	0	0	
2	x, y, z	18,2	B3LYP/6-31G(d,p)	0,1	0	-0,1	0	0	0	
2	-x, y+1/2, -z+1/2	9,75	B3LYP/6-31G(d,p)	0,9	-0,1	-0,9	0	0,1	0,1	
1	-x, -y, -z	12,75	B3LYP/6-31G(d,p)	0,3	0	-0,2	0	0,2	0,1	
2	x, y, z	18,2	B3LYP/6-31G(d,p)	0,2	0	-0,1	0	0,1	0,1	
2	x, y, z	18,5	B3LYP/6-31G(d,p)	0,1	0	-0,1	0	0,1	0,1	
1	-x, -y, -z	18,8	B3LYP/6-31G(d,p)	0,2	0	-0,1	0	0,2	0,1	
2	x, y, z	10,64	B3LYP/6-31G(d,p)	0,7	0	-0,5	0	0,3	0,3	
									-129,55	

Table S20. *Crystal Explorer 21* estimate of lattice energies (kJ/mol) for (*R*)-4-bromophenyl 3-chlorobenzyl sulfoxide **5**

N	Symop	R	Electron Density	E_ele	E_pol	E_dis	E_rep	E_tot		
2	x, y, z	5,68	B3LYP/6-31G(d,p)	-22,9	-6,7	-37,7	30,3	-43,3	-43,3	
2	x+1/2, -y+1/2, -z	7,34	B3LYP/6-31G(d,p)	-5	-0,9	-22,7	13,2	-17,5	-17,5	
2	x+1/2, -y+1/2, -z	7,12	B3LYP/6-31G(d,p)	-4,8	-0,7	-19,6	15,2	-13,4	-13,4	
2	-x, y+1/2, -z+1/2	9,28	B3LYP/6-31G(d,p)	-1,7	-1,3	-20,2	12,9	-12,4	-12,4	
2	-x, y+1/2, -z+1/2	10,87	B3LYP/6-31G(d,p)	-4,1	-1,9	-7,5	6,4	-8,3	-8,3	
2	-x+1/2, -y, z+1/2	10,01	B3LYP/6-31G(d,p)	-1,7	-0,3	-8,1	4,1	-6,6	-6,6	
2	-x+1/2, -y, z+1/2	9,99	B3LYP/6-31G(d,p)	-1,6	-0,4	-8,1	5,9	-5,4	-5,4	-55,3
2	x, y, z	11,37	B3LYP/6-31G(d,p)	-1	0	-0,4	0	-1,5	-1,5	
2	-x, y+1/2, -z+1/2	10,9	B3LYP/6-31G(d,p)	-0,2	-0,1	-1,3	0	-1,4	-1,4	
2	-x+1/2, -y, z+1/2	12,81	B3LYP/6-31G(d,p)	-0,8	0	-0,4	0	-1,2	-1,2	
2	x+1/2, -y+1/2, -z	10,74	B3LYP/6-31G(d,p)	-0,2	0	-0,8	0	-0,8	-0,8	
2	-x, y+1/2, -z+1/2	14,65	B3LYP/6-31G(d,p)	-0,4	0	-0,2	0	-0,7	-0,7	
2	x+1/2, -y+1/2, -z	10,88	B3LYP/6-31G(d,p)	0,1	-0,1	-0,7	0	-0,5	-0,5	
2	x, y, z	12,23	B3LYP/6-31G(d,p)	0	0	-0,6	0	-0,5	-0,5	
2	-x+1/2, -y, z+1/2	12,85	B3LYP/6-31G(d,p)	-0,2	0	-0,3	0	-0,4	-0,4	
2	x, y, z	13,48	B3LYP/6-31G(d,p)	-0,1	0	-0,3	0	-0,3	-0,3	
2	-x, y+1/2, -z+1/2	13,65	B3LYP/6-31G(d,p)	0	0	-0,2	0	-0,2	-0,2	
2	-x, y+1/2, -z+1/2	14,79	B3LYP/6-31G(d,p)	0	0	-0,1	0	-0,2	-0,2	
2	-x, y+1/2, -z+1/2	14,69	B3LYP/6-31G(d,p)	0,1	0	-0,1	0	0	0	
2	-x, y+1/2, -z+1/2	14,77	B3LYP/6-31G(d,p)	0,1	0	-0,1	0	0	0	
2	-x+1/2, -y, z+1/2	15,7	B3LYP/6-31G(d,p)	0,1	0	-0,1	0	0	0	
2	x+1/2, -y+1/2, -z	17,84	B3LYP/6-31G(d,p)	0,1	0	-0,1	0	0	0	
2	-x+1/2, -y, z+1/2	15,71	B3LYP/6-31G(d,p)	0,2	0	-0,1	0	0,1	0,1	
2	x, y, z	16,69	B3LYP/6-31G(d,p)	0,1	0	-0,1	0	0,1	0,1	
2	x+1/2, -y+1/2, -z	17,93	B3LYP/6-31G(d,p)	0,2	0	-0,1	0	0,2	0,2	
2	x, y, z	13,48	B3LYP/6-31G(d,p)	0,5	0	-0,3	0	0,3	0,3	
									-113,9	

Table S21. *Crystal Explorer 21* estimate of lattice energies (kJ/mol) for *rac*-2, 3, 4, 5, 6-pentafluorobenzyl 2, 3, 4, 5, 6-pentafluorophenyl sulfoxide **6**

N	Symop	R	Electron Density	E_ele	E_pol	E_dis	E_rep	E_tot		
2	x, -y, z+1/2	5,39	B3LYP/6-31G(d,p)	-16,7	-3,1	-39,1	21,8	-40,6	-40,6	
2	x, y, z	5,38	B3LYP/6-31G(d,p)	-7,4	-3,6	-45,4	17,1	-39,5	-39,5	
2	x, -y, z+1/2	5,62	B3LYP/6-31G(d,p)	-2,4	-1,3	-31,4	8,9	-25,4	-25,4	-105,5
2	x, -y, z+1/2	13,84	B3LYP/6-31G(d,p)	-2,5	0	-4,5	0	-6,6	-6,6	
2	x, y, z	14,04	B3LYP/6-31G(d,p)	-2,6	0	-2,6	0	-5,1	-5,1	
2	x, -y, z+1/2	13,74	B3LYP/6-31G(d,p)	-1	0	-4,5	0	-5	-5	
2	x, y, z	12,97	B3LYP/6-31G(d,p)	6,3	-0,2	-11,1	0	-3,2	-3,2	
2	x, y, z	10,75	B3LYP/6-31G(d,p)	-0,5	0	-0,6	0	-1,1	-1,1	
2	x, y, z	11	B3LYP/6-31G(d,p)	-0,5	0	-0,4	0	-0,9	-0,9	
2	x, -y, z+1/2	9,6	B3LYP/6-31G(d,p)	0,4	-0,1	-1,2	0	-0,7	-0,7	
2	x, y, z	9,6	B3LYP/6-31G(d,p)	0,3	0	-0,9	0	-0,5	-0,5	
2	x, -y, z+1/2	15,62	B3LYP/6-31G(d,p)	-0,2	0	-0,3	0	-0,5	-0,5	
2	x, -y, z+1/2	14,33	B3LYP/6-31G(d,p)	0,1	0	-0,5	0	-0,4	-0,4	
2	x, -y, z+1/2	15,87	B3LYP/6-31G(d,p)	-0,2	0	-0,2	0	-0,4	-0,4	
2	x, -y, z+1/2	9,18	B3LYP/6-31G(d,p)	1	-0,1	-1,4	0	-0,3	-0,3	
2	x, y, z	16,51	B3LYP/6-31G(d,p)	-0,1	0	-0,2	0	-0,3	-0,3	
2	x, y, z	16,85	B3LYP/6-31G(d,p)	-0,2	0	-0,1	0	-0,3	-0,3	
2	x, -y, z+1/2	16,15	B3LYP/6-31G(d,p)	-0,1	0	-0,1	0	-0,2	-0,2	
2	x, y, z	11	B3LYP/6-31G(d,p)	0,3	0	-0,6	0	-0,1	-0,1	
2	x, y, z	14,42	B3LYP/6-31G(d,p)	0	0	-0,1	0	-0,1	-0,1	
2	x, y, z	15,61	B3LYP/6-31G(d,p)	0,2	0	-0,3	0	0	0	
2	x, y, z	16,51	B3LYP/6-31G(d,p)	0,1	0	-0,1	0	0	0	
2	x, y, z	18,96	B3LYP/6-31G(d,p)	0	0	0	0	0	0	
2	x, y, z	17,49	B3LYP/6-31G(d,p)	0,1	0	-0,1	0	0,1	0,1	
2	x, y, z	16,85	B3LYP/6-31G(d,p)	0,3	0	-0,1	0	0,2	0,2	
2	x, -y, z+1/2	16,39	B3LYP/6-31G(d,p)	0,4	0	-0,1	0	0,3	0,3	
2	x, y, z	14,04	B3LYP/6-31G(d,p)	1,3	0	-0,8	0	0,7	0,7	
2	x, -y, z+1/2	14,42	B3LYP/6-31G(d,p)	1,1	0	-0,5	0	0,7	0,7	
									-129,2	

Table S22. *Crystal Explorer 21* estimate of lattice energies (kJ/mol) for (*R*)-2, 3, 4, 5, 6-pentafluorobenzyl 2, 3, 4, 5, 6-pentafluorophenyl sulfoxide **6**

N	Symop	R	Electron Density	E_ele	E_pol	E_dis	E_rep	E_tot		
2	x, y, z	5,92	B3LYP/6-31G(d,p	-4,1	-0,7	-36,6	11,4	-29,7	-29,7	
2	-x, y+1/2, -z	8,37	B3LYP/6-31G(d,p	-14,3	-3,5	-15,5	10,7	-24,6	-24,6	
2	-x, y+1/2, -z	6,85	B3LYP/6-31G(d,p	-3,4	-0,5	-27,8	8,6	-22,8	-22,8	
2	x, y, z	9,03	B3LYP/6-31G(d,p	-8	-1,4	-19,3	7,6	-21,7	-21,7	
2	-x, y+1/2, -z	8,8	B3LYP/6-31G(d,p	-1,4	-1	-17,5	7,8	-12,6	-12,6	-81,7
2	x, y, z	10,8	B3LYP/6-31G(d,p	0,9	-0,1	-6	0,5	-4	-4	
2	-x, y+1/2, -z	11,14	B3LYP/6-31G(d,p	0,2	-0,1	-2,8	0	-2,3	-2,3	
2	x, y, z	10,8	B3LYP/6-31G(d,p	-0,7	0	-1	0	-1,7	-1,7	
2	-x, y+1/2, -z	12,14	B3LYP/6-31G(d,p	-0,9	0	-0,6	0	-1,4	-1,4	
2	-x, y+1/2, -z	11,84	B3LYP/6-31G(d,p	-0,3	0	-0,5	0	-0,7	-0,7	
2	-x, y+1/2, -z	10,81	B3LYP/6-31G(d,p	0	0	-0,8	0	-0,6	-0,6	
2	-x, y+1/2, -z	11,53	B3LYP/6-31G(d,p	0	0	-0,7	0	-0,6	-0,6	
2	x, y, z	13,56	B3LYP/6-31G(d,p	-0,2	0	-0,2	0	-0,4	-0,4	
2	x, y, z	14,88	B3LYP/6-31G(d,p	-0,2	0	-0,2	0	-0,4	-0,4	
2	-x, y+1/2, -z	14,25	B3LYP/6-31G(d,p	-0,2	0	-0,1	0	-0,3	-0,3	
2	x, y, z	14,8	B3LYP/6-31G(d,p	-0,2	0	-0,1	0	-0,3	-0,3	
2	x, y, z	14,89	B3LYP/6-31G(d,p	-0,1	0	-0,2	0	-0,3	-0,3	
2	x, y, z	11,83	B3LYP/6-31G(d,p	0,1	0	-0,4	0	-0,2	-0,2	
2	x, y, z	14,8	B3LYP/6-31G(d,p	-0,1	0	-0,1	0	-0,2	-0,2	
2	x, y, z	13,67	B3LYP/6-31G(d,p	0,2	0	-0,3	0	-0,1	-0,1	
2	-x, y+1/2, -z	15,74	B3LYP/6-31G(d,p	0	0	-0,2	0	-0,1	-0,1	
2	x, y, z	14,89	B3LYP/6-31G(d,p	0,2	0	-0,1	0	0,1	0,1	
2	-x, y+1/2, -z	13,93	B3LYP/6-31G(d,p	0,4	0	-0,2	0	0,2	0,2	
2	-x, y+1/2, -z	15,03	B3LYP/6-31G(d,p	0,5	0	-0,1	0	0,3	0,3	
									-124,4	

Table S23. *Crystal Explorer 21* estimate of lattice energies (kJ/mol) for *rac*-2-chloro-5-((2, 3, 4, 5, 6-pentafluorophenylsulfinyl)methyl)thiophene **7**

N	Symop	R	Electron Density	E_ele	E_pol	E_dis	E_rep	E_tot		
2	x, y, z	5,14	B3LYP/6-31G(d,p)	-15,8	-5,1	-42,9	24,1	-43	-43	
2	x, -y+1/2, z+1/2	5,15	B3LYP/6-31G(d,p)	-17,4	-3,8	-35,1	23,6	-37,1	-37,1	
2	x, -y+1/2, z+1/2	5,66	B3LYP/6-31G(d,p)	-5,7	-1,6	-29,9	15,9	-23,4	-23,4	-103,5
2	-x, y+1/2, -z+1/2	14,94	B3LYP/6-31G(d,p)	-6,1	-0,1	-4	0	-10	-10	
2	-x, y+1/2, -z+1/2	12,75	B3LYP/6-31G(d,p)	-1,2	0	-4,7	0	-5,4	-5,4	
1	-x, -y, -z	14,29	B3LYP/6-31G(d,p)	1,3	-0,3	-6,3	0	-4,3	-2,15	
1	-x, -y, -z	15	B3LYP/6-31G(d,p)	-1,6	-0,1	-1,9	0	-3,4	-1,7	
1	-x, -y, -z	13,63	B3LYP/6-31G(d,p)	-1	0	-2,2	0	-3	-1,5	
2	x, y, z	10,27	B3LYP/6-31G(d,p)	-1,5	-0,1	-0,6	0	-2,1	-2,1	
1	-x, -y, -z	14,97	B3LYP/6-31G(d,p)	-0,5	0	-0,2	0	-0,7	-0,35	
1	-x, -y, -z	17,19	B3LYP/6-31G(d,p)	-0,5	0	-0,2	0	-0,7	-0,35	
2	x, y, z	10,77	B3LYP/6-31G(d,p)	-0,1	0	-0,4	0	-0,5	-0,5	
1	-x, -y, -z	14,26	B3LYP/6-31G(d,p)	-0,2	0	-0,3	0	-0,5	-0,25	
1	-x, -y, -z	16,57	B3LYP/6-31G(d,p)	-0,2	0	-0,3	0	-0,5	-0,25	
2	-x, y+1/2, -z+1/2	16,61	B3LYP/6-31G(d,p)	-0,3	0	-0,2	0	-0,5	-0,5	
1	-x, -y, -z	17,7	B3LYP/6-31G(d,p)	-0,3	0	-0,1	0	-0,4	-0,2	
2	x, -y+1/2, z+1/2	9,51	B3LYP/6-31G(d,p)	0,8	-0,1	-1,2	0	-0,3	-0,3	
2	x, y, z	13,97	B3LYP/6-31G(d,p)	-0,2	0	-0,1	0	-0,3	-0,3	
2	-x, y+1/2, -z+1/2	14,67	B3LYP/6-31G(d,p)	-0,1	0	-0,3	0	-0,3	-0,3	
1	-x, -y, -z	16,45	B3LYP/6-31G(d,p)	-0,1	0	-0,1	0	-0,3	-0,15	
2	x, -y+1/2, z+1/2	8,59	B3LYP/6-31G(d,p)	1,2	-0,2	-1,5	0	-0,2	-0,2	
1	-x, -y, -z	17,25	B3LYP/6-31G(d,p)	-0,2	0	0	0	-0,2	-0,1	
2	x, y, z	9,47	B3LYP/6-31G(d,p)	0,7	0	-0,9	0	-0,1	-0,1	
2	-x, y+1/2, -z+1/2	17,19	B3LYP/6-31G(d,p)	0	0	-0,1	0	-0,1	-0,1	
1	-x, -y, -z	17,28	B3LYP/6-31G(d,p)	0,1	0	-0,1	0	-0,1	-0,05	
1	-x, -y, -z	17,77	B3LYP/6-31G(d,p)	-0,1	0	-0,1	0	-0,1	-0,05	
1	-x, -y, -z	19,21	B3LYP/6-31G(d,p)	-0,1	0	0	0	-0,1	-0,05	
2	x, y, z	10,77	B3LYP/6-31G(d,p)	0,4	0	-0,5	0	0	0	
1	-x, -y, -z	15,34	B3LYP/6-31G(d,p)	0,1	0	-0,1	0	0	0	
1	-x, -y, -z	15,76	B3LYP/6-31G(d,p)	0,2	0	-0,1	0	0,1	0,05	
1	-x, -y, -z	17,91	B3LYP/6-31G(d,p)	0,2	0	-0,1	0	0,1	0,05	
1	-x, -y, -z	15,37	B3LYP/6-31G(d,p)	0,6	0	-0,5	0	0,2	0,1	
2	-x, y+1/2, -z+1/2	15,58	B3LYP/6-31G(d,p)	0,6	0	-0,3	0	0,3	0,3	
2	-x, y+1/2, -z+1/2	15,99	B3LYP/6-31G(d,p)	0,4	0	-0,1	0	0,3	0,3	
2	-x, y+1/2, -z+1/2	14,24	B3LYP/6-31G(d,p)	0,9	0	-0,4	0	0,6	0,6	
1	-x, -y, -z	13,22	B3LYP/6-31G(d,p)	1,3	0	-0,8	0	0,7	0,35	
2	x, -y+1/2, z+1/2	13,18	B3LYP/6-31G(d,p)	0,9	0	-0,1	0	0,8	0,8	
1	-x, -y, -z	12,41	B3LYP/6-31G(d,p)	8,8	-0,1	-9	0	1,3	0,65	
									-127,25	

Table S24. *Crystal Explorer 21* estimate of lattice energies (kJ/mol) for (*R*)-2-chloro-5-((2, 3, 4, 5, 6-pentafluorophenylsulfinyl)methyl)thiophene **7**

N	Symop	R	Electron Density	E_ele	E_pol	E_dis	E_rep	E_tot		
2	x, y, z	5,43	B3LYP/6-31G(d,p)	-26	-7,5	-38,6	33,8	-45,7	-45,7	
2	-x, y+1/2, -z	7,32	B3LYP/6-31G(d,p)	-2,8	-0,7	-23,1	8,4	-18,3	-18,3	
2	-x, y+1/2, -z	8,21	B3LYP/6-31G(d,p)	-5,5	-1,3	-19,4	11,1	-16,8	-16,8	
2	-x, y+1/2, -z	8,96	B3LYP/6-31G(d,p)	-1	-0,3	-10,7	2,5	-9	-9	
2	-x, y+1/2, -z	9,14	B3LYP/6-31G(d,p)	-4	-1	-12,5	11,2	-8,9	-8,9	
2	-x, y+1/2, -z	9,27	B3LYP/6-31G(d,p)	-0,5	-0,1	-1,5	0	-1,9	-1,9	
2	x, y, z	9,95	B3LYP/6-31G(d,p)	-1,7	-0,2	-9,2	7,7	-5,2	-5,2	-60,1
2	-x, y+1/2, -z	10,51	B3LYP/6-31G(d,p)	0,5	-0,1	-1	0	-0,4	-0,4	
2	x, y, z	10,86	B3LYP/6-31G(d,p)	-1,6	-0,1	-0,5	0	-2,2	-2,2	
2	x, y, z	11,34	B3LYP/6-31G(d,p)	1	-0,1	-0,7	0	0,4	0,4	
2	x, y, z	11,34	B3LYP/6-31G(d,p)	-0,4	-0,1	-4,6	1,9	-3,3	-3,3	
2	x, y, z	11,88	B3LYP/6-31G(d,p)	0,3	0	-0,6	0	-0,2	-0,2	
2	x, y, z	12,43	B3LYP/6-31G(d,p)	0,8	0	-0,5	0	0,4	0,4	
2	-x, y+1/2, -z	12,59	B3LYP/6-31G(d,p)	-0,2	-0,1	-0,4	0	-0,6	-0,6	
2	-x, y+1/2, -z	12,88	B3LYP/6-31G(d,p)	0,2	0	-0,3	0	-0,1	-0,1	
2	-x, y+1/2, -z	13,32	B3LYP/6-31G(d,p)	0	0	-0,1	0	-0,1	-0,1	
2	x, y, z	13,67	B3LYP/6-31G(d,p)	0,4	0	-0,1	0	0,3	0,3	
2	x, y, z	14,73	B3LYP/6-31G(d,p)	0	0	-0,1	0	-0,1	-0,1	
2	x, y, z	14,73	B3LYP/6-31G(d,p)	-0,6	0	-0,2	0	-0,8	-0,8	
2	x, y, z	15,05	B3LYP/6-31G(d,p)	0,2	0	-0,1	0	0,1	0,1	
2	x, y, z	15,5	B3LYP/6-31G(d,p)	0,5	0	-0,1	0	0,5	0,5	
2	-x, y+1/2, -z	15,86	B3LYP/6-31G(d,p)	-0,1	0	-0,1	0	-0,2	-0,2	
2	x, y, z	15,92	B3LYP/6-31G(d,p)	0,2	0	-0,1	0	0,1	0,1	
2	-x, y+1/2, -z	16,29	B3LYP/6-31G(d,p)	-0,3	0	-0,1	0	-0,4	-0,4	
2	-x, y+1/2, -z	16,68	B3LYP/6-31G(d,p)	-0,2	0	-0,1	0	-0,3	-0,3	
2	-x, y+1/2, -z	16,78	B3LYP/6-31G(d,p)	-0,1	0	-0,1	0	-0,2	-0,2	
2	x, y, z	18,04	B3LYP/6-31G(d,p)	0	0	-0,1	0	0	0	
									-112,9	

Table S25. *Crystal Explorer 21* estimate of lattice energies (kJ/mol) for *rac*-2-(2, 3, 4, 5, 6-pentafluorobenzyl)sulfinyl thiophene **8**.

N	Symop	R	Electron Density	E_ele	E_pol	E_dis	E_rep	E_tot		
2	x, y, z	5,35	B3LYP/6-31G(d,p)	-20,5	-5,1	-39,7	32,1	-40,2	-40,2	-40,2
1	-x, -y, -z	7,43	B3LYP/6-31G(d,p)	-39,5	-8,6	-23,1	42,4	-42	-21	
2	-x, y+1/2, -z+1/2	7,42	B3LYP/6-31G(d,p)	-6,8	-1,7	-23,6	17,8	-18	-18	
1	-x, -y, -z	7,81	B3LYP/6-31G(d,p)	-8,2	-1	-19,3	7,7	-21,4	-10,7	
2	x, y, z	8,53	B3LYP/6-31G(d,p)	1,4	-0,7	-19,3	8,7	-10,5	-10,5	
2	x, y, z	10,07	B3LYP/6-31G(d,p)	-3,2	-0,6	-9,7	6,5	-8,2	-8,2	
1	-x, -y, -z	8,17	B3LYP/6-31G(d,p)	0,6	-1,4	-11,6	5,8	-6,9	-3,45	
2	-x, y+1/2, -z+1/2	9,46	B3LYP/6-31G(d,p)	0,5	-0,1	-3	0,1	-2,1	-2,1	
2	x, y, z	10,69	B3LYP/6-31G(d,p)	-1,4	-0,1	-0,5	0	-2	-2	
2	x, y, z	10,07	B3LYP/6-31G(d,p)	-0,3	-0,1	-1,2	0	-1,5	-1,5	
1	-x, -y, -z	8,52	B3LYP/6-31G(d,p)	0,5	-0,2	-2,9	0	-2,1	-1,05	-75
2	x, -y+1/2, z+1/2	13,46	B3LYP/6-31G(d,p)	-0,8	0	-0,1	0	-1	-1	
2	-x, y+1/2, -z+1/2	10,6	B3LYP/6-31G(d,p)	-0,2	-0,1	-0,8	0	-0,9	-0,9	
2	x, y, z	13,68	B3LYP/6-31G(d,p)	-0,6	0	-0,2	0	-0,8	-0,8	
2	x, -y+1/2, z+1/2	15,01	B3LYP/6-31G(d,p)	-0,7	0	-0,1	0	-0,8	-0,8	
2	x, -y+1/2, z+1/2	12,81	B3LYP/6-31G(d,p)	-0,4	0	-0,2	0	-0,7	-0,7	
1	-x, -y, -z	13,96	B3LYP/6-31G(d,p)	-1,1	0	-0,2	0	-1,4	-0,7	
1	-x, -y, -z	14,37	B3LYP/6-31G(d,p)	-0,8	0	-0,2	0	-1,1	-0,55	
2	x, -y+1/2, z+1/2	14,43	B3LYP/6-31G(d,p)	-0,2	0	-0,1	0	-0,4	-0,4	
2	-x, y+1/2, -z+1/2	12,11	B3LYP/6-31G(d,p)	0	0	-0,3	0	-0,2	-0,2	
2	x, -y+1/2, z+1/2	14,29	B3LYP/6-31G(d,p)	0,1	0	-0,1	0	-0,1	-0,1	
1	-x, -y, -z	14,58	B3LYP/6-31G(d,p)	-0,1	0	-0,1	0	-0,2	-0,1	
1	-x, -y, -z	16,07	B3LYP/6-31G(d,p)	0	0	-0,1	0	-0,1	-0,05	
1	-x, -y, -z	10,33	B3LYP/6-31G(d,p)	1,5	-0,2	-1,6	0	0	0	
2	-x, y+1/2, -z+1/2	16,41	B3LYP/6-31G(d,p)	0,1	0	-0,1	0	0	0	
1	-x, -y, -z	11,89	B3LYP/6-31G(d,p)	0,3	0	-0,3	0	0,1	0,05	
1	-x, -y, -z	16,59	B3LYP/6-31G(d,p)	0,2	0	-0,1	0	0,1	0,05	
2	x, y, z	13,68	B3LYP/6-31G(d,p)	0,2	0	-0,1	0	0,1	0,1	
2	-x, y+1/2, -z+1/2	14,95	B3LYP/6-31G(d,p)	0,4	0	-0,1	0	0,2	0,2	
2	x, -y+1/2, z+1/2	15,76	B3LYP/6-31G(d,p)	0,3	0	-0,1	0	0,2	0,2	
1	-x, -y, -z	14,47	B3LYP/6-31G(d,p)	0,7	0	-0,1	0	0,7	0,35	
1	-x, -y, -z	11,64	B3LYP/6-31G(d,p)	1,4	-0,1	-0,5	0	1	0,5	
2	-x, y+1/2, -z+1/2	12,9	B3LYP/6-31G(d,p)	1,6	-0,1	-0,9	0	0,9	0,9	
1	-x, -y, -z	10,04	B3LYP/6-31G(d,p)	5,3	-0,4	-0,9	0	4,5	2,25	
									-120,4	

Table S26. *Crystal Explorer 21* estimate of lattice energies (kJ/mol) for (*R*)-2-(2, 3, 4, 5, 6-pentafluorobenzyl)sulfinyl thiophene **8**.

N	Symop	R	Electron Density	E_ele	E_pol	E_dis	E_rep	E_tot		
2	x, y, z	5,49	B3LYP/6-31G(d,p)	-18,1	-4,5	-36,4	25,4	-38,4	-38,4	
2	-x, y+1/2, -z+1/2	7,19	B3LYP/6-31G(d,p)	-5,3	-0,7	-20,4	8,8	-18,5	-18,5	
2	-x, y+1/2, -z+1/2	7,59	B3LYP/6-31G(d,p)	-2,6	-0,6	-16,9	6	-14,2	-14,2	
2	x+1/2, -y+1/2, -z	9,17	B3LYP/6-31G(d,p)	-2,3	-1,3	-18,7	12,6	-12	-12	
2	x+1/2, -y+1/2, -z	8,06	B3LYP/6-31G(d,p)	-3,5	-3,3	-13,3	11,2	-10,9	-10,9	
2	x, y, z	10,73	B3LYP/6-31G(d,p)	-1,5	-0,2	-2	0,1	-3,5	-3,5	
2	x, y, z	9,22	B3LYP/6-31G(d,p)	2,3	-0,7	-7,5	1,9	-3,4	-3,4	-59
2	x+1/2, -y+1/2, -z	11,19	B3LYP/6-31G(d,p)	-2,2	-0,1	-0,5	0	-2,9	-2,9	
2	x, y, z	10,99	B3LYP/6-31G(d,p)	-1,3	-0,1	-0,4	0	-1,7	-1,7	
2	-x, y+1/2, -z+1/2	10,3	B3LYP/6-31G(d,p)	-0,6	0	-0,9	0	-1,4	-1,4	
2	x+1/2, -y+1/2, -z	12,02	B3LYP/6-31G(d,p)	-0,6	-0,1	-0,6	0	-1,2	-1,2	
2	x, y, z	14,34	B3LYP/6-31G(d,p)	-1	0	-0,1	0	-1,2	-1,2	
2	-x+1/2, -y, z+1/2	11,79	B3LYP/6-31G(d,p)	-0,3	0	-0,8	0	-1	-1	
2	-x, y+1/2, -z+1/2	15,08	B3LYP/6-31G(d,p)	-0,4	0	-0,1	0	-0,5	-0,5	
2	-x+1/2, -y, z+1/2	12,78	B3LYP/6-31G(d,p)	0	0	-0,4	0	-0,4	-0,4	
2	-x+1/2, -y, z+1/2	14,31	B3LYP/6-31G(d,p)	-0,2	0	-0,1	0	-0,4	-0,4	
2	-x, y+1/2, -z+1/2	14,88	B3LYP/6-31G(d,p)	-0,3	0	-0,1	0	-0,4	-0,4	
2	-x+1/2, -y, z+1/2	15,59	B3LYP/6-31G(d,p)	-0,3	0	-0,1	0	-0,4	-0,4	
2	-x+1/2, -y, z+1/2	15,14	B3LYP/6-31G(d,p)	-0,2	0	-0,1	0	-0,3	-0,3	
2	-x+1/2, -y, z+1/2	16,35	B3LYP/6-31G(d,p)	-0,1	0	-0,1	0	-0,2	-0,2	
2	x, y, z	14,34	B3LYP/6-31G(d,p)	0	0	-0,1	0	-0,1	-0,1	
2	-x+1/2, -y, z+1/2	15,75	B3LYP/6-31G(d,p)	0	0	-0,1	0	-0,1	-0,1	
2	-x, y+1/2, -z+1/2	11,13	B3LYP/6-31G(d,p)	0,4	0	-0,5	0	0	0	
2	-x+1/2, -y, z+1/2	13,24	B3LYP/6-31G(d,p)	0,2	0	-0,2	0	0	0	
2	-x+1/2, -y, z+1/2	15,52	B3LYP/6-31G(d,p)	0,1	0	-0,1	0	0	0	
2	x+1/2, -y+1/2, -z	17,39	B3LYP/6-31G(d,p)	0,2	0	0	0	0,2	0,2	
2	x, y, z	10,73	B3LYP/6-31G(d,p)	1	-0,1	-0,7	0	0,3	0,3	
2	x+1/2, -y+1/2, -z	14,68	B3LYP/6-31G(d,p)	0,4	0	-0,1	0	0,4	0,4	
2	x+1/2, -y+1/2, -z	16,52	B3LYP/6-31G(d,p)	0,5	0	-0,1	0	0,5	0,5	
									-111,7	

Table S27. *Crystal Explorer 21* estimate of lattice energies (kJ/mol) for *rac*-2,4-dichlorophenyl 2, 3, 4, 5, 6-pentafluorobenzyl sulfoxide **9**

N	Symop	R	Electron Density	E_ele	E_pol	E_dis	E_rep	E_tot		
2	x, y, z	7,89	B3LYP/6-31G(d,p)	-15,3	-3,5	-24,3	22,2	-26,1	-26,1	
2	-x+1/2, y+1/2, -z+1/2	6,94	B3LYP/6-31G(d,p)	-3,2	-1	-36,9	18,8	-24,6	-24,6	
2	-x+1/2, y+1/2, -z+1/2	8,35	B3LYP/6-31G(d,p)	-8,7	-2,2	-15,8	8,4	-19,4	-19,4	
1	-x, -y, -z	8,03	B3LYP/6-31G(d,p)	-10,9	-1,2	-22	20,8	-18,6	-9,3	
2	x+1/2, -y+1/2, z+1/2	9,71	B3LYP/6-31G(d,p)	-1,4	-0,2	-6,6	2,8	-5,7	-5,7	
2	x+1/2, -y+1/2, z+1/2	9	B3LYP/6-31G(d,p)	-0,8	-0,3	-6	1,1	-5,6	-5,6	
1	-x, -y, -z	8,84	B3LYP/6-31G(d,p)	-4	-0,3	-17,6	13,9	-11,1	-5,55	
1	-x, -y, -z	7,12	B3LYP/6-31G(d,p)	0,2	-0,9	-17,2	7,4	-10,8	-5,4	-101,65
2	x+1/2, -y+1/2, z+1/2	11,39	B3LYP/6-31G(d,p)	-0,4	0	-1,3	0	-1,6	-1,6	
1	-x, -y, -z	11,62	B3LYP/6-31G(d,p)	-1	-0,1	-0,8	0	-1,8	-0,9	
1	-x, -y, -z	10,89	B3LYP/6-31G(d,p)	-1,9	-0,1	-4,8	7,7	-1,5	-0,75	
2	-x+1/2, y+1/2, -z+1/2	12,3	B3LYP/6-31G(d,p)	0,1	0	-0,7	0	-0,5	-0,5	
2	x+1/2, -y+1/2, z+1/2	15,24	B3LYP/6-31G(d,p)	-0,3	0	-0,2	0	-0,5	-0,5	
2	x+1/2, -y+1/2, z+1/2	15,52	B3LYP/6-31G(d,p)	-0,3	0	-0,1	0	-0,5	-0,5	
2	x+1/2, -y+1/2, z+1/2	11,96	B3LYP/6-31G(d,p)	0,2	0	-0,6	0	-0,3	-0,3	
2	x, y, z	15,06	B3LYP/6-31G(d,p)	-0,1	0	-0,2	0	-0,3	-0,3	
1	-x, -y, -z	12,9	B3LYP/6-31G(d,p)	0	0	-0,5	0	-0,4	-0,2	
1	-x, -y, -z	15,17	B3LYP/6-31G(d,p)	-0,1	0	-0,2	0	-0,3	-0,15	
2	x, y, z	12,82	B3LYP/6-31G(d,p)	0,2	0	-0,3	0	-0,1	-0,1	
2	x+1/2, -y+1/2, z+1/2	13,87	B3LYP/6-31G(d,p)	0,1	0	-0,2	0	-0,1	-0,1	
1	-x, -y, -z	13,92	B3LYP/6-31G(d,p)	-0,1	0	-0,1	0	-0,2	-0,1	
2	-x+1/2, y+1/2, -z+1/2	15,1	B3LYP/6-31G(d,p)	0	0	-0,1	0	-0,1	-0,1	
2	x, y, z	15,78	B3LYP/6-31G(d,p)	0	0	-0,1	0	-0,1	-0,1	
1	-x, -y, -z	12,16	B3LYP/6-31G(d,p)	0,3	0	-0,5	0	-0,1	-0,05	
1	-x, -y, -z	16,23	B3LYP/6-31G(d,p)	0	0	-0,1	0	-0,1	-0,05	
1	-x, -y, -z	13,42	B3LYP/6-31G(d,p)	0,3	0	-0,2	0	0,1	0,05	
1	-x, -y, -z	17,24	B3LYP/6-31G(d,p)	0,2	0	-0,1	0	0,1	0,05	
2	x, y, z	14,38	B3LYP/6-31G(d,p)	0,2	0	-0,2	0	0,1	0,1	
2	-x+1/2, y+1/2, -z+1/2	14,69	B3LYP/6-31G(d,p)	0,3	0	-0,2	0	0,2	0,2	
2	x, y, z	15,57	B3LYP/6-31G(d,p)	0,3	0	-0,1	0	0,2	0,2	
									-107,4	

Table S28. *Crystal Explorer 21* estimate of lattice energies (kJ/mol) for (*R*)-2,4-dichlorophenyl 2, 3, 4, 5, 6-pentafluorobenzyl sulfoxide **9**

N	Symop	R	Electron Density	E_ele	E_pol	E_dis	E_rep	E_tot		
2	x+1/2, -y+1/2, -z	6,46	B3LYP/6-31G(d,p)	-17,4	-4	-33,5	20,1	-38,1	-38,1	
2	-x, y+1/2, -z+1/2	7,07	B3LYP/6-31G(d,p)	-2,5	-0,8	-30,5	13,5	-21,5	-21,5	
2	-x+1/2, -y, z+1/2	8,93	B3LYP/6-31G(d,p)	-2,4	-0,4	-17,3	6	-14,2	-14,2	
2	x, y, z	7,7	B3LYP/6-31G(d,p)	-4,3	-0,8	-17,9	12	-13,3	-13,3	
2	-x+1/2, -y, z+1/2	9,32	B3LYP/6-31G(d,p)	-0,6	-0,2	-7,8	1,9	-6,5	-6,5	
2	-x, y+1/2, -z+1/2	10,1	B3LYP/6-31G(d,p)	-0,6	-0,1	-5,1	1,8	-4	-4	-97,6
2	x+1/2, -y+1/2, -z	11,79	B3LYP/6-31G(d,p)	0,1	-0,2	-5,7	4,5	-2,3	-2,3	
2	-x, y+1/2, -z+1/2	10,8	B3LYP/6-31G(d,p)	0,2	-0,1	-1,3	0	-1	-1	
2	x+1/2, -y+1/2, -z	11,26	B3LYP/6-31G(d,p)	0,2	0	-1,2	0	-0,9	-0,9	
2	-x+1/2, -y, z+1/2	12,33	B3LYP/6-31G(d,p)	-0,1	0	-0,6	0	-0,7	-0,7	
2	-x+1/2, -y, z+1/2	12,62	B3LYP/6-31G(d,p)	-0,2	0	-0,5	0	-0,7	-0,7	
2	x, y, z	12,55	B3LYP/6-31G(d,p)	-0,3	0	-0,3	0	-0,6	-0,6	
2	x+1/2, -y+1/2, -z	12,66	B3LYP/6-31G(d,p)	-0,1	0	-0,3	0	-0,4	-0,4	
2	x, y, z	15,07	B3LYP/6-31G(d,p)	-0,2	0	-0,2	0	-0,4	-0,4	
2	x, y, z	15,41	B3LYP/6-31G(d,p)	-0,2	0	-0,1	0	-0,3	-0,3	
2	x+1/2, -y+1/2, -z	15,66	B3LYP/6-31G(d,p)	-0,1	0	-0,1	0	-0,2	-0,2	
2	-x, y+1/2, -z+1/2	15,69	B3LYP/6-31G(d,p)	-0,1	0	-0,1	0	-0,2	-0,2	
2	x, y, z	16,92	B3LYP/6-31G(d,p)	-0,1	0	-0,1	0	-0,2	-0,2	
2	-x+1/2, -y, z+1/2	13,82	B3LYP/6-31G(d,p)	0,1	0	-0,3	0	-0,1	-0,1	
2	-x, y+1/2, -z+1/2	13,4	B3LYP/6-31G(d,p)	0,3	0	-0,4	0	0	0	
2	-x+1/2, -y, z+1/2	14,59	B3LYP/6-31G(d,p)	0,1	0	-0,2	0	0	0	
2	-x+1/2, -y, z+1/2	16,23	B3LYP/6-31G(d,p)	0,1	0	-0,1	0	0	0	
2	x, y, z	16,92	B3LYP/6-31G(d,p)	0,1	0	-0,1	0	0	0	
2	x, y, z	14,72	B3LYP/6-31G(d,p)	0,2	0	-0,1	0	0,1	0,1	
2	-x, y+1/2, -z+1/2	15,22	B3LYP/6-31G(d,p)	0,3	0	-0,1	0	0,1	0,1	
2	x+1/2, -y+1/2, -z	16,05	B3LYP/6-31G(d,p)	0,2	0	-0,1	0	0,1	0,1	
2	x, y, z	14,72	B3LYP/6-31G(d,p)	0,3	0	-0,1	0	0,2	0,2	
									-105,1	

Table S29. Characteristics of main hydrogen bondings in not previously reported sulfoxides.

Crystal Structure		Angle (°)	H···O (Å)	C···O (Å)	H-C (Å)
rac-**2**	C25-H25-O2	134.7	2.54	3.266(3)	0.93
rac-**3**	C25-H25-O2	145.4	2.29	3.098(4)	0.93
rac-**4**	C1-H1B-O1	170.7	2.38	3.351(5)	0.98
rac-**5**	C22-H22-O2	154.4	2.49	3.349(4)	0.93
(*R*)-**7**	C1-H1B-O2	157.8	2.33	3.254(5)	0.97
rac-**8**	C1-H1A-O1	155.2	2.38	3.293(4)	0.98
rac-**9**	C23-H23-O2	169.8	2.28	3.198(4)	0.93

Table S30. Characteristics of halogen bondings in not previously reported sulfoxides.

Crystal Structure		Angle (°)	Br···O (Å)	C···O (Å)	Br-C (Å)
rac-**3**	C24-Br24-O18	171.7(1)	3.217(4)	5.104(5)	1.899(3)
rac-**4**	C11-Br1-O1	175.6(2)	3.037(3)	4.937(5)	1.903(4)

Table S31. Summary of cell dimensions of the aryl benzyl sulfoxides **1-9** (in Å)

Compound	*a* (Å)	*b* (Å)	*c* (Å)
rac-**1**	8.8256(4)	5.470(3)	23.995(13)
(*R*)-**1**	5.6721(4)	11.4044(8)	17.1743(12)
rac-**2**	7.3042(5)	7.6512(5)	12.3586(8)
(*R*)-**2**	7.7200(10)	11.6763(12)	7.7771(10)
rac-**3**	7.5989(5)	13.6591(8)	13.3151(8)
(*R*)-**3**	5.5750(4)	13.5510(5)	18.1990(11)
rac-**4**	5.5795(3)	15.8297(7)	15.0311(6)
(*R*)-**4**	5.647(4)	13.31(2)	18.064(11)
rac-**5**	14.7677(9)	10.6376(7)	8.7815(5)
(*R*)-**5**	5.6825(13)	12.229(2)	19.178(5)
rac-**6**	12.966(3)	5.3774(11)	9.6002(19)
(*R*)-**6**	9.0321(9)	5.9155(6)	13.5634(14)
rac-**7**	26.479(3)	5.1357(4)	9.4706(8)
(*R*)-**7**	5.4298(5)	9.9501(8)	11.8841(10)
rac-**8**	8.5319(3)	5.3467(2)	25.5242(8)
(*R*)-**8**	5.4947(11)	9.2154(18)	23.473(5)
rac-**9**	7.8924(7)	12.8239(11)	14.3783(12)
(*R*)-**9**	7.7040(10)	12.545(2)	15.068(3)

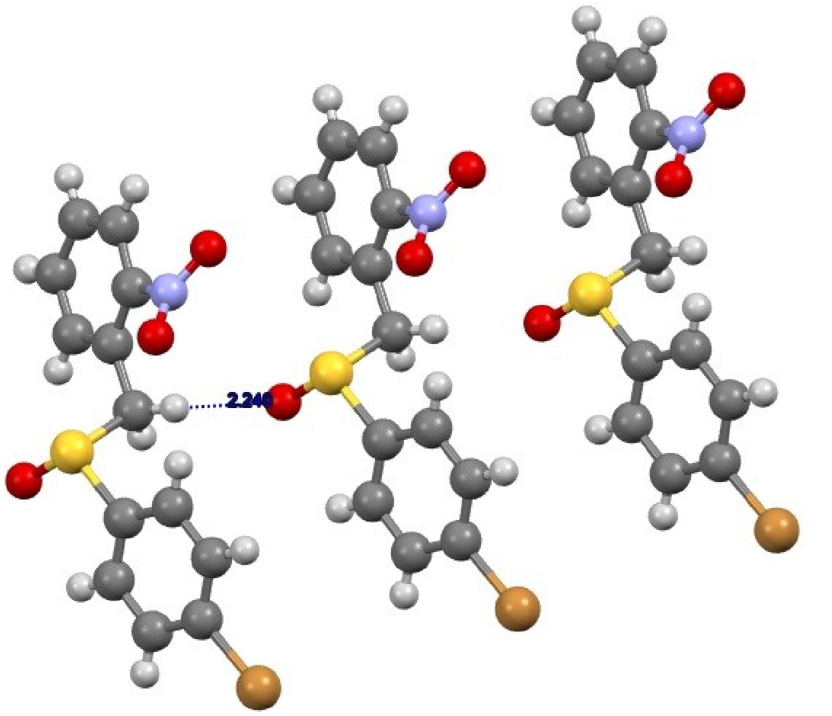

Figure S21. Representation of the action of the “Displace” operator in *anti*-sulfoxide (*R*)-**3.**

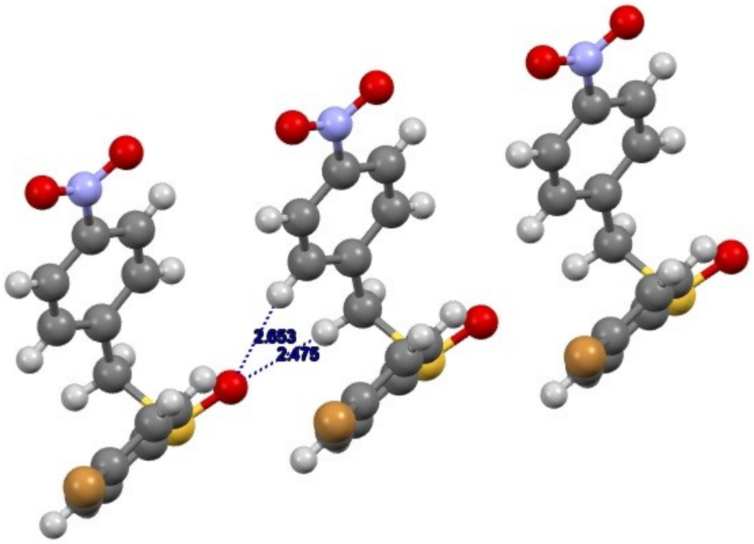

Figure S22. Representation of the action of the “Displace” operator in *gauche*-sulfoxide (*R*)-**4.**

Graphical Reports of spectral data

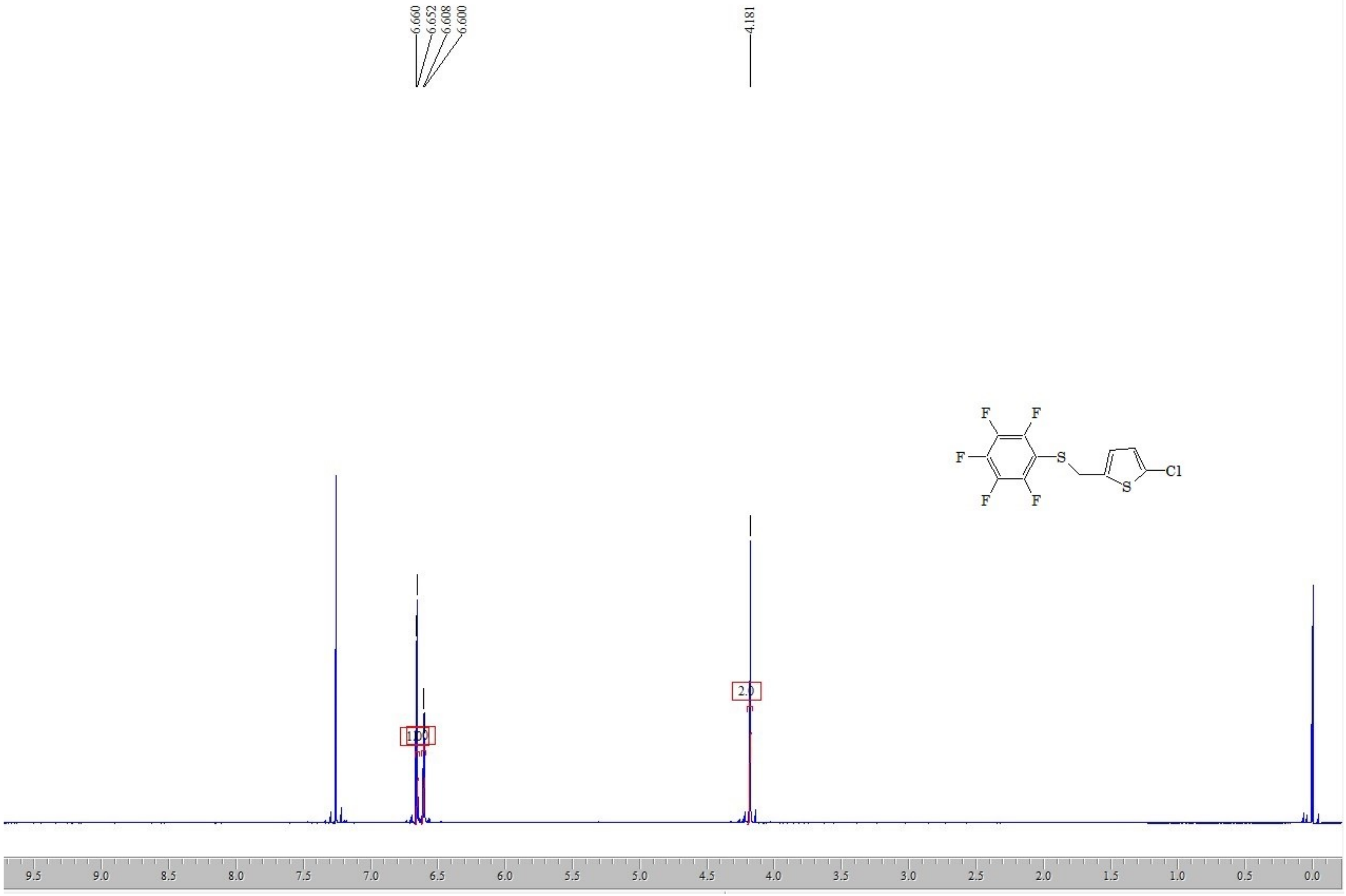

Figure S23. 500 MHz ^{1}H-NMR spectrum of 2-chloro-5-((2, 3, 4, 5, 6-pentafluorophenylthio)methyl)thiophene.

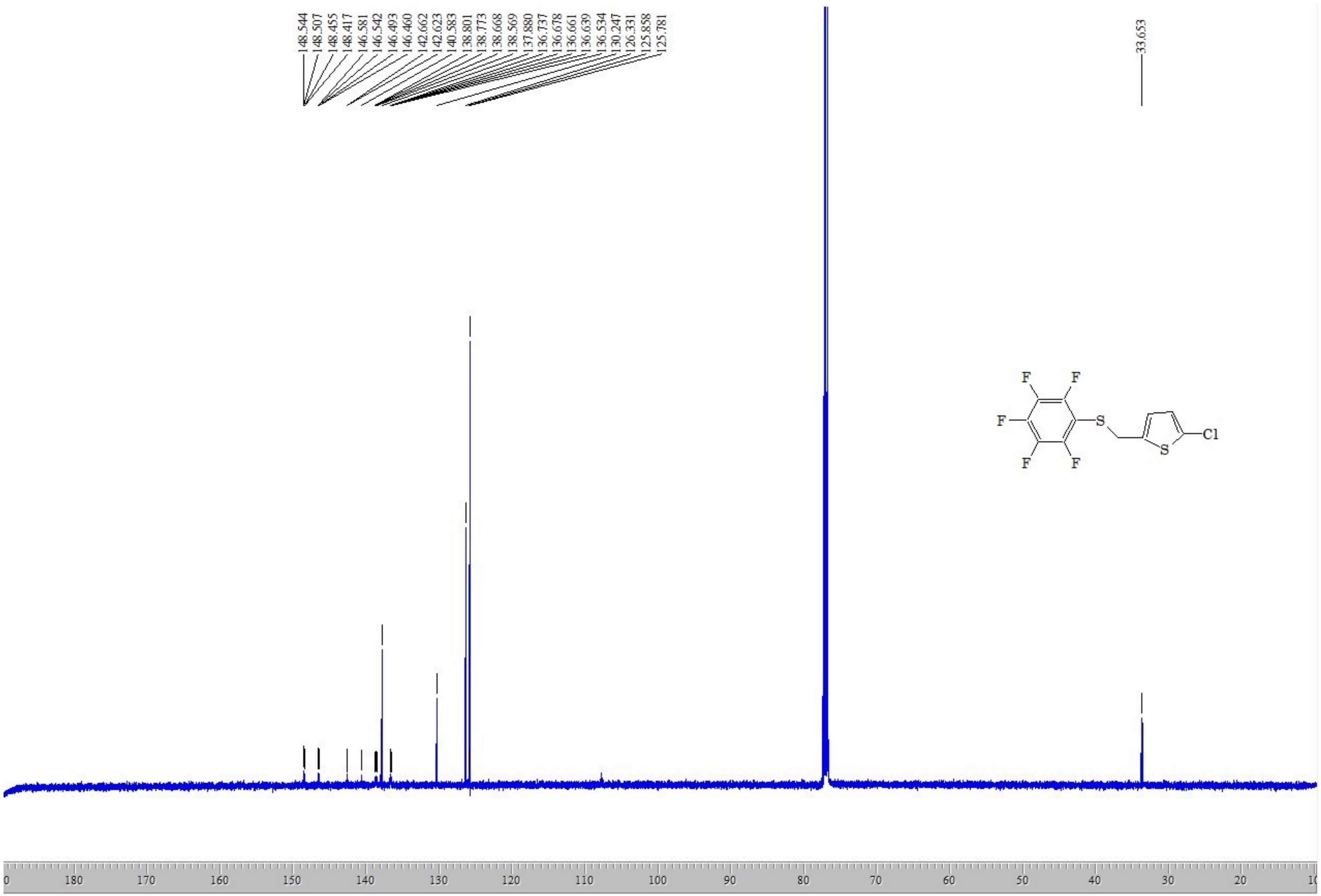

Figure S24. 125 MHz ^{13}C-NMR spectrum of 2-chloro-5-((2, 3, 4, 5, 6-pentafluorophenylthio)methyl)thiophene.

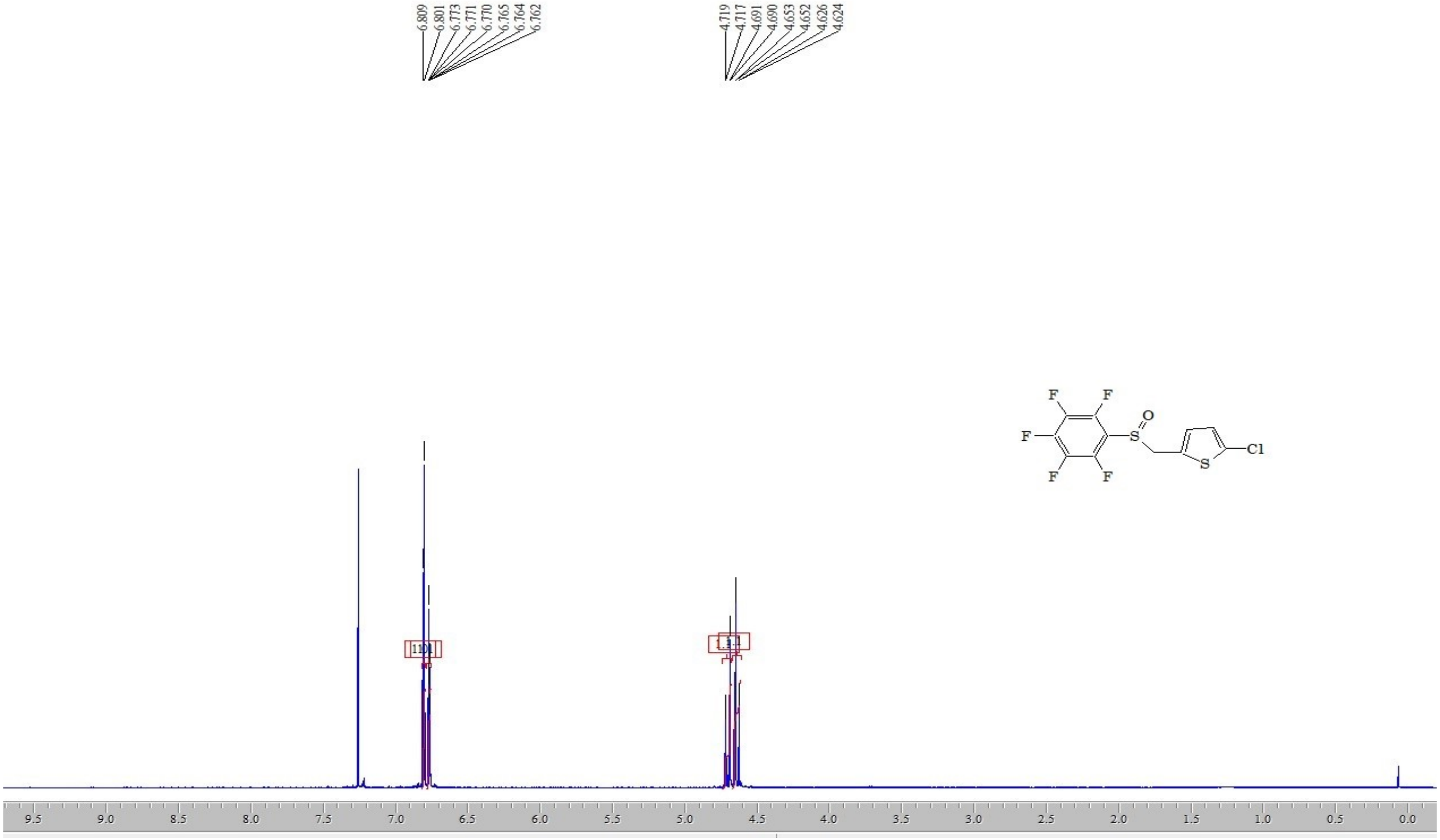

Figure S25. 500 MHz ^{1}H-NMR spectrum of 2-chloro-5-((2, 3, 4, 5, 6-pentafluorophenylsulfinyl)methyl)thiophene **7.**

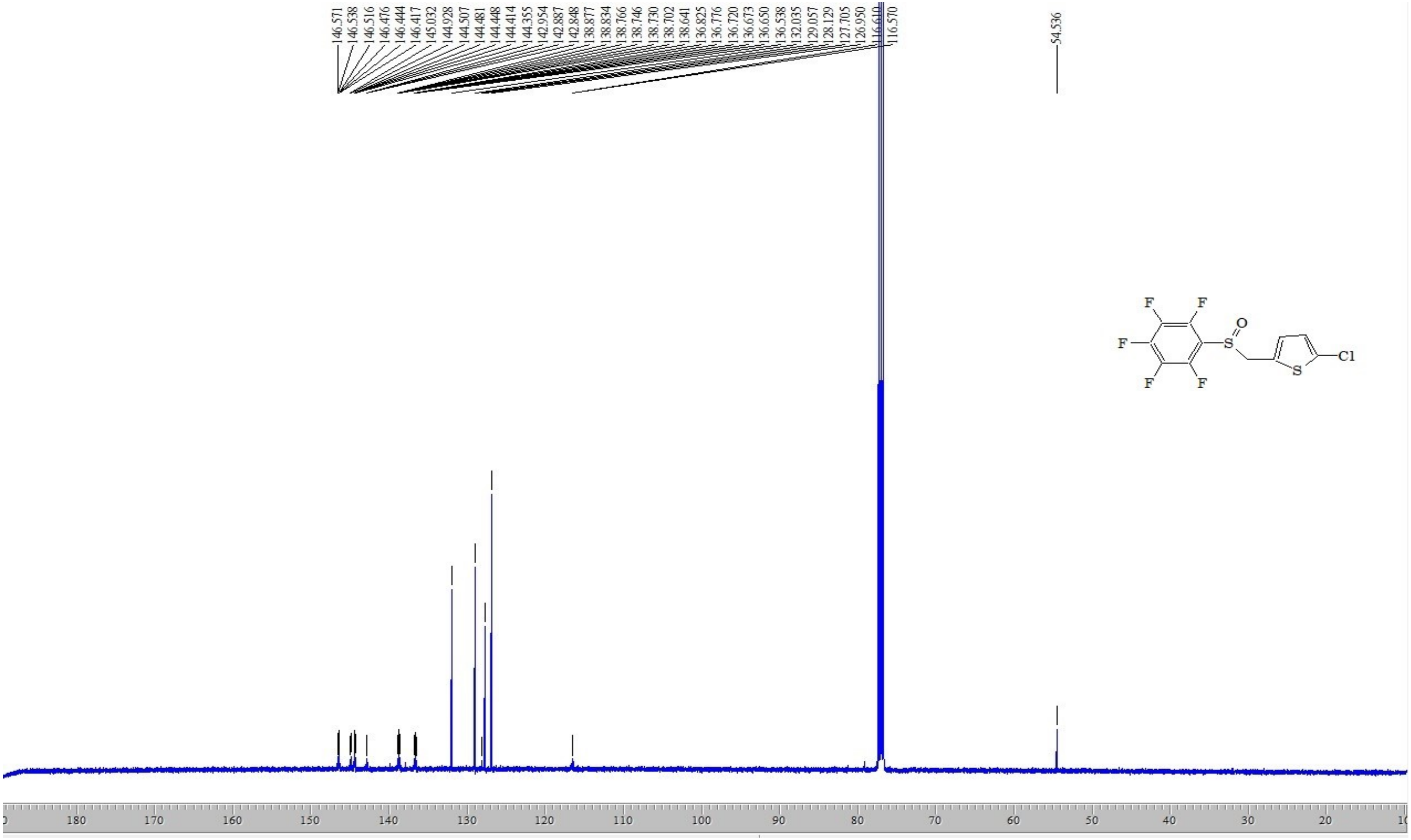

Figure S26. 125 MHz ^{13}C-NMR spectrum of 2-chloro-5-((2, 3, 4, 5, 6-pentafluorophenylsulfinyl)methyl)thiophene **7.**
